# Digital behavioral addictions in adolescents and young adults: Protocol for a scoping review of definitions, measurement, epidemiology, determinants, harms, interventions, and equity

**DOI:** 10.1371/journal.pone.0348860

**Published:** 2026-09-11

**Authors:** Sasha R. Sioni, Jenna L. Butner, Nathan A. Carroll, Jay Al-Hashimi, Sten H. Vermund

**Affiliations:** 1 Health Management and Policy, Patti & Allan Herbert Business School, University of Miami, Miami, Florida, United States of America; 2 Department of Medical Education, Herbert Wertheim College of Medicine, Florida International University, Miami, Florida, United States of America; 3 Department of Internal Medicine (General Medicine), Yale University School of Medicine, New Haven, Connecticut, United States of America; 4 Department of Psychiatry, Jersey Shore University Medical Center, Hackensack Meridian Health, Neptune City, New Jersey, United States of America; 5 The Warren Alpert Medical School of Brown University, Providence, Rhode Island, United States of America; 6 College of Public Health, USF Health, University of South Florida, Tampa, Florida, United States of America; Ministry of Health, Sri Lanka, SRI LANKA

## Abstract

**Background:**

Digital behaviors associated with impaired control and functional impairment are described using heterogeneous terms and measures. Gaming Disorder is recognized in ICD-11, Internet Gaming Disorder remains a DSM-5-TR condition for further study, and problematic social media and smartphone use are research constructs rather than established diagnoses. These distinctions avoid equating screening-positive use with clinical disorder.

**Objective:**

This scoping review will map definitions, measurement, epidemiology, determinants, harms, interventions, implementation, and equity among adolescents and young adults.

**Methods:**

Following JBI guidance, we will search nine bibliographic and grey-literature databases and supplementary sources from inception, without database-level date or language limits. The core population is aged 10–24 years; separately extractable data for ages 25–29 will form a secondary comparison stratum, while inseparable broader-age samples will be used only for contextual purposes. Two reviewers will independently screen and chart. Automation will not make eligibility, appraisal, synthesis, or interpretation decisions; frameworks have bounded module-specific roles. Every final eligibility decision and extracted finding from six eligible non-English languages will undergo source-language human verification. Instruments will be classified per use into graded evidence tiers without tier-based exclusion. Result-level rules will distinguish determinants, concurrent associations, harms, intervention effectiveness, adverse events, and implementation outcomes. All seven module-specific charting forms will be completed before calibration, and module-stratified calibration will precede full charting. Primary and secondary evidence will remain separate, and overlap will be tracked through study, report, sample, review, and result identifiers. Qualitative findings will undergo dual-coded framework analysis. Source-level appraisal will be descriptive and non-exclusionary. No confirmatory hypothesis testing or meta-analysis is planned.

**Expected contribution:**

The review will produce construct- and instrument-specific evidence maps and an equity-oriented gap map intended to inform measurement selection, service and policy planning, intervention development, and priorities for focused systematic reviews. Registration: https://doi.org/10.17605/OSF.IO/NPWTV

## Introduction

Digital technologies are embedded in young people’s social, educational, and recreational lives. A subset of users report impaired control, increasing priority given to a digital activity, persistence despite adverse consequences, or functional impairment. The terminology applied to these experiences is not uniform and does not represent a single diagnostic category. Gaming Disorder is recognized in ICD-11, whereas Internet Gaming Disorder is retained in DSM-5-TR as a condition for further study [1–3]. Problematic social media use and problematic smartphone use are research constructs with variable definitions and thresholds [4–6]. In this review, “digital behavioral addictions” is therefore a pragmatic search and mapping label, not a claim of nosological equivalence or a statement that all screening-positive behavior constitutes disorder.

Evidence is distributed across syntheses of separate constructs and methodological questions. Construct-specific reviews address prevalence [7–12], psychometric measurement [13,14], determinants [15–17], harms such as sleep, aggression, and broader health outcomes [18–20], and interventions [21,22]. Differences in diagnostic systems, instruments, language adaptations, cutoffs, sampling frames, and age bands impede comparison. The same variable may also be modeled as an antecedent, a concurrent correlate, a later outcome, or an intervention endpoint. Intervention research adds distinctions among effectiveness, adverse events, and implementation, while equity-relevant characteristics are inconsistently reported. A shared map is needed to link these domains without collapsing their conceptual differences.

The primary population is aged 10–24 years, consistent with an expanded developmental framing of adolescence and common policy and indexing bands [23–29]. Separately extractable data for ages 25–29 will be retained only as a secondary comparison stratum because prominent emerging-adulthood scholarship extends through age 29 [30]. Inseparable broader-age samples will not support age-specific quantitative conclusions.

A scoping review is appropriate because the objectives are to clarify concepts, describe how research has been conducted, map heterogeneous evidence, and identify gaps rather than estimate one pooled effect or grade a narrowly defined evidence body [31–34]. A focused systematic review would address a narrower question, whereas an umbrella review would be restricted to existing reviews and would not directly map eligible primary evidence.

Date-bounded searches of OSF Registries, PROSPERO, INPLASY, and JBI Evidence Synthesis on July 12, 2026, identified several related protocols addressing individual construct families or domains; however, we did not identify a publicly accessible, independent protocol that combined all four target construct families, all seven prespecified evidence domains, and the 10–29-year age scope. This finding is limited by differences in registry eligibility and indexing, including PROSPERO’s exclusion of scoping reviews, INPLASY’s archive-page search behavior, and the status of JBI Evidence Synthesis as a publication corpus rather than a prospective registry. Exact queries, records, and limitations are reported in S1 Appendix. No claim of being the first protocol is made.

Following JBI guidance, the review will address seven linked questions concerning definitions and classification, measurement, epidemiology, determinants, harms, interventions and implementation, and equity. The practical aim is to produce transparent construct- and instrument-specific maps that can inform measurement selection, service and policy planning, intervention development, and priorities for subsequent focused systematic reviews.

## Materials and methods

### Study design, reporting, and registration

This scoping review will be conducted in accordance with JBI methodology [32–34] and reported using the PRISMA Extension for Scoping Reviews [35]. Protocol reporting follows PRISMA-P [36,37], as required by PLOS ONE for scoping-review Study Protocols; the completed checklist is provided as S1 Checklist. The protocol is registered at OSF Registries (https://doi.org/10.17605/OSF.IO/NPWTV). Because the submitted registration is immutable, current working materials, future review data, and versioned supporting files will be maintained in the associated public OSF project (https://osf.io/rav8m/). Substantive departures from the registered plan will be dated and justified in S3 Appendix and submitted as a formal OSF registration update where applicable.

As of July 13, 2026, the MEDLINE strategy is a draft and PRESS review has not been completed. No formal bibliographic database search, screening, data charting, critical appraisal, or synthesis has begun. Preparatory registry searches undertaken to contextualize novelty do not constitute the formal review search.

### Objectives and analytic orientation

The review addresses descriptive mapping questions and does not posit a confirmatory hypothesis. All analyses are exploratory and will describe the extent, characteristics, and distribution of eligible evidence. No null-hypothesis testing, meta-analysis, effect pooling, or certainty-of-evidence grading is planned.

### Conceptual organization

The seven modules are coordinated organizational structures rather than components of one causal theory. Definitions and classification will preserve distinctions among recognized diagnoses, conditions for further study, and problematic-use constructs. Measurement findings will be organized using COSMIN-defined properties and the per-use evidence tiers specified below [38,39]. Determinants will be mapped to I-PACE categories without treating I-PACE as proof of causal direction [40–42]. Intervention descriptions will use TIDieR [43], implementation outcomes will use Proctor’s taxonomy [44], and equity-related reporting will use PROGRESS-Plus and SAGER [45–49]. Table 1 limits each framework to its intended role.

**Table 1:** Coordinated module architecture.

RQ	Module	Organizing framework or rule	Evidence mapped	Planned output
1	Definitions and classification	Tiered nosological taxonomy	Terms, diagnostic status, core features, impairment, duration, and classification alignment	Terminology and classification map
2	Measurement	COSMIN-defined properties plus per-use evidence tiers	Instrument properties, language or population adaptation, invariance, cutoffs, and interpretability	Instrument-by-property-and-tier matrix
3	Epidemiology	Instrument-, threshold-, tier-, and age-specific presentation	Diagnostic prevalence, screen-positive prevalence, denominators, and subgroups	Stratified epidemiological matrices
4	Determinants	I-PACE categories plus result-role and temporality rules	Prospective determinants, concurrent modeled predictors, mediators, and moderators	Direction-annotated determinant matrix
5	Harms	Outcome domains plus result-role and temporality rules	Concurrent and prospective mental, sleep, physical, social, academic, and neurocognitive outcomes	Direction-annotated harms table
6	Interventions and implementation	TIDieR and Proctor	Intervention components, effectiveness, adverse events, and implementation outcomes	Intervention-effectiveness-implementation matrix
7	Equity	PROGRESS-Plus and SAGER	Representation, subgroup analysis, accessibility, and reporting gaps	Equity table and evidence-gap map

### Review questions

Seven linked questions are presented in Table 2. Questions 1–4 address definitions and classification, measurement, epidemiology, and determinants; Questions 5–6 concern harms, interventions, and implementation; Question 7 is cross-cutting.

**Table 2:** Review questions.

RQ	Module	Question
1	Definitions and classification	How are Gaming Disorder, Internet Gaming Disorder, problematic social media use, and problematic smartphone use defined and classified in evidence relevant to adolescents and young adults?
2	Measurement	What measurement properties, language or population adaptations, cutoffs, and evidence tiers are reported for instruments used with these constructs?
3	Epidemiology	What diagnostic or screen-positive prevalence estimates are reported for the core 10–24 population, and how do estimates vary by instrument, threshold, region, sex or gender, and age band?
4	Determinants	Which factors precede, predict, mediate, moderate, or occur concurrently with the target constructs, and how are they mapped to I-PACE categories?
5	Harms	Which adverse outcomes are reported in association with the target constructs, and what do study design and temporality permit regarding direction?
6	Interventions and implementation	Which interventions have been evaluated, what effectiveness and adverse-event outcomes are reported, and what implementation outcomes are described?
7	Equity	How are PROGRESS-Plus characteristics and sex or gender addressed, and where are reporting or evidence gaps apparent?

Table 2 note: Separately extractable evidence for ages 25–29 will be reported as a secondary comparison stratum and will not be merged with core 10–24 evidence.

### Eligibility criteria

#### Population and age rules.

The core population comprises participants aged 10–24 years. A source may contribute core evidence only when all analyzed participants are aged 10–24 or when results for that range are separately extractable. Separately extractable results for ages 25–29 will be retained as a secondary comparison stratum and reported separately from core evidence.

A sample mean or median within the target range will never establish core or secondary eligibility. Results from an inseparable broader-age sample will be classified as contextual evidence from a mixed-age sample and may contribute only to definitions, instrument-use history, qualitative context, secondary-review context, or implementation description. Such samples will not contribute age-specific psychometric properties, prevalence, determinants, harms, equity comparisons, effectiveness estimates, or other quantitative conclusions about people aged 10–24 or 25–29. An inseparable sample spanning ages 18–29 is therefore contextual rather than core or secondary evidence. A mixed-age source with neither separately extractable age data nor an eligible contextual contribution will be excluded.

Each charted sample or result will be classified as core evidence for ages 10–24, secondary comparison evidence for ages 25–29, contextual evidence from a mixed-age sample, or excluded. A report may therefore receive more than one classification across separately extractable samples or age strata. At the source level, a report will be included when it has at least one eligible contribution and excluded only when it has none. If both a total-sample estimate and an eligible subgroup estimate are reported, only the subgroup estimate will enter the applicable age-specific output.

#### Concepts and construct boundaries.

Eligible target constructs are ICD-11 Gaming Disorder, DSM-5-TR Internet Gaming Disorder, problematic social media use, and problematic smartphone use [1–6]. Related labels may be eligible when the source clearly identifies one target behavior and reports separately extractable findings. Online gambling, problematic pornography use, other non-target behavioral addictions, ordinary high-frequency use, and undifferentiated Internet or screen use without a named target behavior are outside scope.

Use of the Internet Addiction Test, Compulsive Internet Use Scale, or another generic Internet-use instrument does not by itself establish eligibility [50]. Such evidence is eligible only when the instrument is explicitly applied to a named target behavior and findings for that behavior are separately extractable.

#### Measurement-evidence tiers.

Measurement evidence will be classified for each instrument use; tiers are not permanent labels attached to an instrument.

- **Tier 1:** formal DSM or ICD diagnostic criteria, or a standardized instrument with published validation evidence in the study language and a relevant adolescent or young-adult population.
- **Tier 2:** a standardized instrument with published validation evidence in another population or language, or an instrument that underwent only partial translation or adaptation for the present use.
- **Tier 3:** an ad hoc measure, single item, unstructured judgment, or instrument for which no published validation evidence can be identified for that use.
- **N/A:** no measure was used, as may occur in a conceptual source or guideline.

Tier assignment will use a prespecified search for validation evidence for each instrument use. Two reviewers will examine the source report and its cited validation references, the instrument manual or developer website when available, PubMed, PsycINFO, and the COSMIN database of systematic reviews of outcome measurement instruments. Searches will combine the exact instrument name or acronym with terms for the study language, age group, and validation or measurement properties. The search log will record the date, exact search strings, identifiers, population and language match, and tier decision. Tier 1 or Tier 2 requires an identifiable validation source; if the search does not identify an applicable validation source, the instrument use will be classified as Tier 3. Disagreements will be resolved by adjudication, and tier changes will be versioned.

No source will be excluded solely because of measurement tier. Diagnostic prevalence will be reported only when ascertainment used formal diagnostic criteria administered through an appropriate diagnostic procedure. Estimates based on scale thresholds will be labeled “screen-positive prevalence” or the source authors’ more cautious equivalent. Tier 3 findings may describe reported or endorsed problematic use but will never be labeled disorder prevalence.

#### Context, dates, and languages.

No geographic region, country-income category, or clinical, community, school, university, household, or online setting will be excluded. “All regions” denotes the absence of a geographic eligibility restriction; it does not remove the language limitation below.

**Fig 1:**
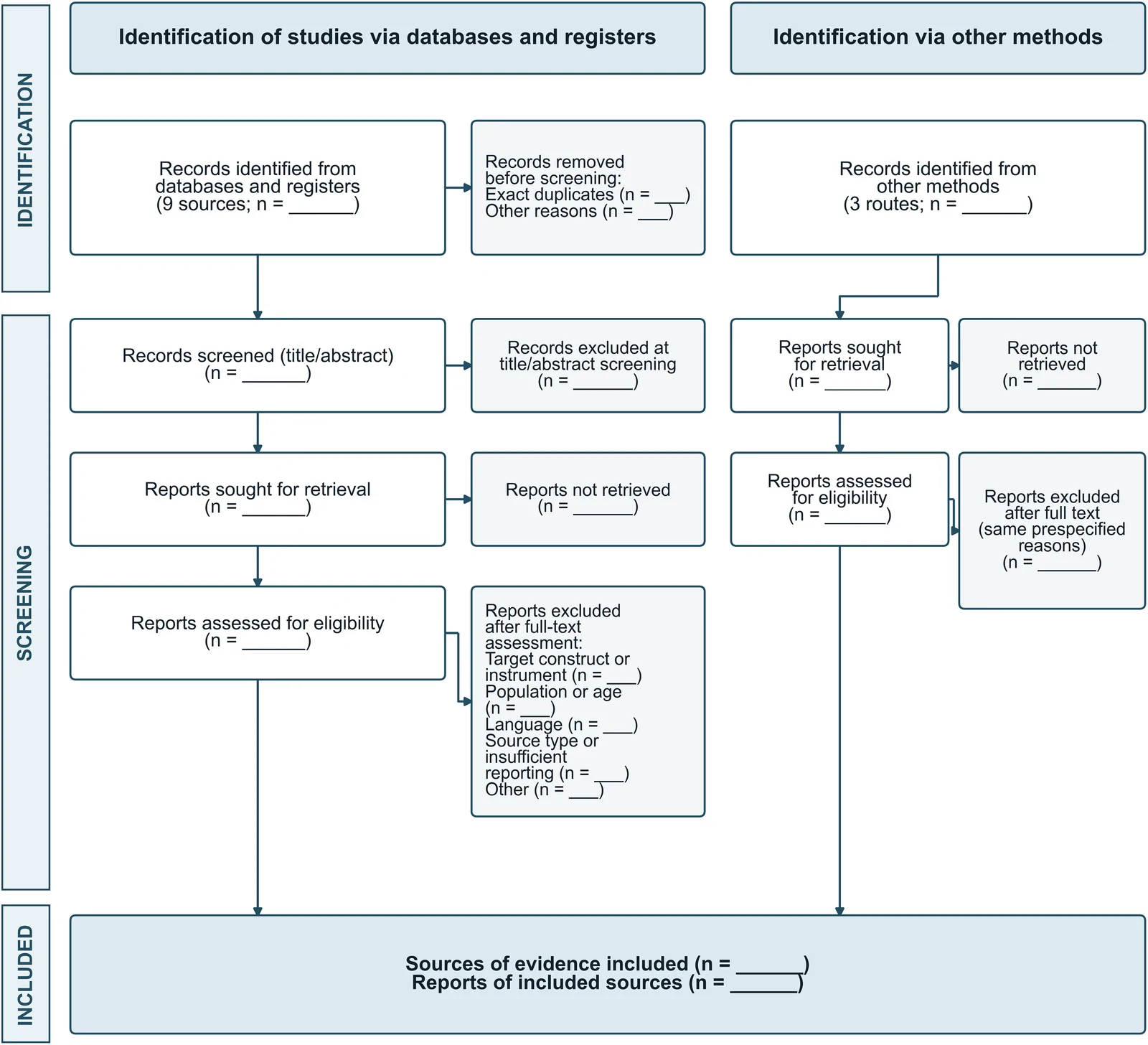
PRISMA 2020 flow diagram template for the planned review. This blank two-lane template will be populated after searching, screening, and study selection. ProQuest Dissertations & Theses Global and WHO Global Index Medicus are classified as databases; Google Scholar, websites and organizations, and backward and forward citation searching are classified as other methods. Full-text exclusion reasons reflect the prespecified eligibility criteria. Publication date is not an exclusion criterion. Adapted from the PRISMA 2020 flow diagram [59], distributed under the Creative Commons Attribution 4.0 International (CC BY 4.0) license (https://creativecommons.org/licenses/by/4.0/).

Databases will be searched from inception. No publication-date restriction will be applied in the search or during screening. Dates associated with diagnostic recognition, manual editions, instrument development, or policy adoption will be charted as historical descriptors and may be used for stratified presentation; they will not determine eligibility or appear as exclusion reasons in Fig 1.

Records in English, Spanish, French, German, Portuguese, Arabic, and Hebrew are eligible. No database-level language filter will be applied. Records in other languages will be excluded only after publication language has been identified during screening, and language-specific exclusion counts will be reported. Machine translation evidence from particular language pairs cannot establish reliability for a different language or task [51,52]; therefore, the human-verification procedure below applies to every final non-English decision and finding.

#### Evidence types.

Eligible primary evidence includes quantitative, qualitative, mixed-methods, intervention, implementation, and psychometric studies. Systematic reviews, meta-analyses, and scoping reviews are eligible as secondary evidence but will be charted and synthesized separately from primary studies. Official classifications, guidelines, and consensus statements are eligible only when retrieved through the prespecified database, grey-literature, or citation-searching pathways; they will provide contextual information and will not be combined with research outputs. Ad hoc surveillance briefs and standalone policy or implementation documents are not formal sources of evidence.

Editorials, letters without original data, opinion pieces, narrative commentaries, book chapters without original data, and conference abstracts are excluded. Full peer-reviewed conference papers or proceedings are eligible when they otherwise meet the criteria.

Table 3 consolidates the boundary rules used at screening and charting.

**Table 3:** Eligibility and boundary rules.

Domain	Included	Excluded	Boundary rule
Core population	Entire analyzed sample aged 10–24, or separately extractable 10–24 results	No eligible age-specific or contextual contribution	Mean or median age alone never establishes core eligibility
Secondary age stratum	Separately extractable 25–29 results	Inseparable broader samples presented as 25–29 evidence	Report separately from core evidence
Mixed-age context	Definitions, instrument-use history, qualitative context, secondary-review context, or implementation description	Age-specific psychometric, prevalence, determinant, harm, equity, or effectiveness conclusions	Classify as contextual evidence from a mixed-age sample; an inseparable sample spanning ages 18–29 is contextual
Constructs	Gaming Disorder, Internet Gaming Disorder, problematic social media use, and problematic smartphone use	Gambling, pornography use, undifferentiated Internet overuse, or ordinary high-frequency use without a target problem construct	A related label is eligible only when a target behavior and its findings are separately identifiable
Instruments	Tiers 1–3 and N/A	None excluded solely by tier	Generic IAT or CIUS use is eligible only when applied to a named target behavior with separately extractable findings
Prevalence terminology	Formal diagnostic prevalence or scale-based screen-positive prevalence	Tier 3 estimates labeled disorder prevalence	Preserve source ascertainment and use cautious labels
Dates	Database inception onward	No publication-year exclusion	Diagnostic and instrument milestones are historical strata only
Languages	English, Spanish, French, German, Portuguese, Arabic, and Hebrew	Other languages	No database filter; exclude during screening and enumerate by language
Evidence	Primary research; systematic reviews or meta-analyses; scoping reviews; prespecified-pathway official classifications, guidelines, or consensus statements	Editorials, opinions, narrative commentaries, ad hoc surveillance briefs, and standalone policy or implementation documents	Source levels are charted and synthesized separately
Conference material	Full peer-reviewed proceedings or papers	Abstract-only records	A full report must permit eligibility assessment and charting
Setting	Clinical, community, school or university, household, or online; all regions	None by setting or geography	The language restriction remains applicable

### Information sources and search strategy

The bibliographic and grey-literature database search will include MEDLINE via Ovid, PsycINFO via Ovid, CINAHL via EBSCO, Scopus, Web of Science Core Collection, the Cochrane Library, IEEE Xplore, ProQuest Dissertations & Theses Global, and WHO Global Index Medicus. Prespecified supplementary methods will include Google Scholar, a targeted search limited to six organizations, and backward and forward citation searching. The organizational pathway is limited to WHO/ICD, the American Psychiatric Association and PsychiatryOnline, UNICEF, UNESCO, the International Telecommunication Union, and NICE. For each listed domain, six exact phrase queries will be run as logged-out site-restricted web searches; all results will be screened when 50 or fewer are returned and otherwise the first 50 in relevance order will be screened. The complete draft MEDLINE strategy and all supplementary-source procedures are reported in S1 Appendix and will be deposited in the associated OSF project before screening [33,53].

The principal database strategy will combine target-construct and instrument terms with population terms. The MEDLINE draft combines the current Internet Addiction Disorder heading and legacy Behavior, Addictive indexing with relevant technology headings and broad free-text variants for addiction, dependence, dysregulation, maladaptive or pathological use, overuse, and problematic or compulsive use. The population block includes the un-exploded Adult heading and the adult* free-text term to protect recall for the 25–29 comparison stratum; some records involving older adults will therefore be retrieved and resolved during screening. Measurement, prevalence, determinant, harm, intervention, qualitative, implementation, and equity terms will not form a mandatory third concept block because requiring them could omit evidence relevant to the seven questions. Searches will run from database inception without date or language limits, consistent with sensitivity-oriented search guidance [54].

Three prespecified Google Scholar queries will address gaming, social-media, and smartphone constructs. For each query, the first 200 results in relevance order will be screened, following Haddaway et al. [55]. Searches will be conducted without an authenticated Google account; the exact query, date and time, displayed result count, browser and session conditions, number screened, and retained citation details will be logged. Incognito mode will be used to reduce account-history effects, not claimed to eliminate personalization. Google Scholar is supplementary and will not replace database or targeted organizational searching.

Backward reference-list searching and forward cited-reference searching will be conducted for all included sources using TARCiS terminology [56,57]. Dates, platforms, and results will be logged. The registry searches described in the Introduction will be updated immediately before formal search execution and before resubmission if the package is delayed; exact strings and limitations will be retained in S1 Appendix.

Before execution, the MEDLINE strategy will undergo PRESS peer review by an independent information specialist [58]. Resulting changes will be recorded in S3 Appendix, incorporated into every database translation, and included in the OSF update. No claim is made that a librarian or information specialist has already reviewed the strategy.

### Record management and study selection

Search results will be imported into Covidence and assigned stable source identifiers. Exact duplicates matched on stable identifiers may be collapsed automatically, with each action retained in the audit log. Every non-exact or near-duplicate cluster will require human confirmation; ambiguous clusters will be checked by a second reviewer. No automated function will exclude a record on substantive eligibility grounds.

Two reviewers will independently screen every title and abstract. Before full screening, they will assess a 50-record calibration set supplemented as needed to represent target constructs and evidence types. Full screening will begin after percent agreement reaches at least 80%; Cohen’s kappa will be reported when calculable. If the threshold is not met, eligibility guidance will be clarified and tested on 50 new records. Any substantive rule change will be versioned and recorded in S3 Appendix.

Two reviewers will then independently assess full texts. A ten-report full-text calibration exercise will precede full assessment. Disagreements at either stage will be resolved through discussion and, if necessary, adjudication by a third reviewer. One primary exclusion reason will be recorded for each excluded full text using the hierarchy in Table 3. Selection will be reported in the PRISMA 2020 flow diagram (Fig 1) [59].

### Human verification of non-English evidence

The team has documented access to source-language-proficient reviewers for Spanish, French, German, Portuguese, Arabic, and Hebrew. If that capacity becomes unavailable before or during the review, the affected work will pause until coverage is restored or the protocol is formally amended.

Machine translation may be used to prepare a working rendering, but no eligibility decision, exclusion reason, charted value, interpretation, or quotation will rest solely on machine output. For every record in an eligible non-English language, a source-language-proficient person will check the original for each final title-and-abstract and full-text decision. A second reviewer will assess the human-verified rendering when making the independent decision.

For charting, the source-language reviewer will verify construct terminology, eligibility-relevant wording, direction of association, numerical values, uncertainty measures, qualitative findings, and any quotation against the original. The second reviewer will chart from the verified rendering and source locator. The translation log will record language, verifier, stated proficiency, machine tool and version if used, date, fields checked, discrepancies, and corrections. English-language evidence will undergo the same dual human screening and charting process but will not require translation verification.

### Data-charting forms and fields

Charting will use Pollock’s dual-table structure: Table A defines variables, accepted values, decision rules, critical fields, and hypothetical examples; Table B contains populated records [32,60]. S2 Appendix provides complete Table A forms for all seven modules before calibration. No module form is deferred for development during full charting.

Each retrieved document will be assigned unique source and report identifiers. Primary research will also receive a study identifier and, for each distinct analyzed sample or wave, a sample identifier; secondary reviews will receive a review identifier. A dataset-family identifier and any other applicable identifiers will be assigned only when relevant and otherwise coded N/A. Core fields include citation, country, setting, design, sample size, age distribution, sample- or result-level age-based evidence classification, sex and gender reporting, source language, source level, target construct, instrument and version, measurement tier, and applicable module assignments. Each charted quantitative or qualitative finding will receive a unique result identifier. Result fields include construct, instrument use and tier, population stratum, primary and linked modules, result role, temporality, direction, estimate, uncertainty, adjustment status, source locator, whether the field is designated critical, translation provenance, and overlap links.

The initial charting forms contain 32 core fields, including a source-level appraisal method. Before calibration, the initial core form and all seven module-specific forms will be version-locked. Applicable module forms contain 12 fields for definitions and classification, 22 for measurement, 15 for epidemiology, 13 for determinants, 12 for harms, 26 for interventions and implementation, and 14 for equity; the separate qualitative-integration matrix and citation matrix linking included reviews to their cited primary studies contain nine and six fields, respectively. A source completes one core record and only its applicable module and result rows, so workload grows with the number of reported samples, instruments, and results; no source requires completion of every field. During calibration, reviewers will log minutes per source–module pair, applicable and missing field counts, reconciliation time, and reviewer assignment. These observations will determine workload allocation and any timeline revision before full charting. If adequate dual-reviewer capacity cannot be sustained, charting will pause and any staged output or scope change will be documented as a protocol amendment before work resumes.

#### Module-specific charting.

**Definitions and classification:** source terminology; stated nosological status; classification system; definitional approach; core features; functional-impairment and duration requirements; categorical or dimensional framing; citation source; and relationship to established diagnostic criteria.

**Measurement:** instrument name and version; administration mode; target construct; respondent and population; study language; translation or adaptation; per-use tier; content validity; structural validity; internal consistency; cross-cultural validity or invariance; reliability; measurement error; criterion validity; hypotheses testing for construct validity; responsiveness where applicable; cutoff derivation; feasibility; and interpretability [38,39]. COSMIN criteria will be applied only to properties and instrument types for which they are applicable. Internal consistency will be interpreted only when structural validity or unidimensionality is adequate. Structural validity will use the alternative COSMIN v2.0 criteria rather than a conjunctive CFI-and-RMSEA rule. The protocol will not reduce properties to one binary validated or unvalidated judgment. Every example in S2 Appendix is explicitly hypothetical.

**Epidemiology:** target construct; ascertainment method; measurement tier; diagnostic procedure or scale threshold; denominator; sample frame; diagnostic prevalence or screen-positive frequency; confidence interval; country or region; sex or gender; and age stratum. Estimates will remain separated by instrument, threshold, tier, and population stratum. Common instruments and classifications will be recorded without presuming comparability [50,61,62].

**Determinants:** putative determinant; I-PACE category where applicable; design; exposure and outcome timing; model specification; adjusted and unadjusted estimate; confounders; mediator or moderator status; direction; and result-role classification. I-PACE coding will organize reported factors without converting concurrent evidence into causal determinants.

**Harms:** outcome domain and measure; timing relative to the target construct; concurrent or prospective design; adjusted and unadjusted estimate; confounders; direction; and result-role classification. Prospective direction will be distinguished from cross-sectional association.

**Interventions and implementation:** the 12 TIDieR items; comparator; allocation and follow-up; target-construct outcome; intended secondary outcomes; effect estimates and uncertainty; unintended adverse events; and Proctor implementation outcomes of acceptability, adoption, appropriateness, feasibility, fidelity, implementation cost, penetration, and sustainability [43,44].

**Equity:** PROGRESS-Plus characteristics; sex and gender terminology and analysis under SAGER; population inclusion and exclusion; subgroup analyses; accessibility or adaptation; reporting gaps; and whether an observed difference is descriptive, adjusted, or explicitly analyzed as an inequity [45–49]. Absence of reporting will not be interpreted as absence of inequity.

### Charting calibration and full charting

Before full charting, two reviewers will independently chart at least five study-module pairs for each of the seven modules and at least ten unique reports overall. The same report may contribute to more than one module. If fewer than five eligible pairs are available for a module, every available pair will be used and the small denominator reported.

The calibration denominator includes every jointly applicable structured categorical field. NR, NA, and UNCLEAR are categorical decisions: disagreement between either of these codes and another code or value, or between two different missingness codes, counts as disagreement. A field is excluded from the denominator only when both reviewers independently select NA and reconciled applicability confirms that the field does not apply. The gate requires at least 90% agreement overall and at least 80% agreement within each module and for every critical field with at least one applicable comparison. Continuous numerical values and source locators will be compared exactly and reconciled separately rather than included in the categorical denominator. Cohen’s kappa will be reported for suitable categorical fields when calculable but will not replace the percent-agreement gates.

If a module or critical field fails, its guidance and hypothetical examples will be revised and tested on five new study–module pairs, or all remaining new pairs if fewer than five exist. Earlier records affected by a rule change will be re-charted under the new codebook version. A critical field with no applicable paired ratings will be recorded as not estimable, not passed. Reviewers will add eligible calibration pairs until the field has an applicable comparison; if every available pair is jointly and correctly NA, a third reviewer will confirm the applicability decision, the field will be labeled not applicable in calibration, and calibration will reopen before further charting when the first applicable source appears. Full charting will begin only after every estimable gate passes and each field with no applicable paired ratings has followed this prespecified deferral procedure.

After calibration, two reviewers will independently chart every included source into its applicable modules. Disagreements will be resolved by consensus and, when necessary, third-reviewer adjudication.

### Result-role classification and cross-module overlap

Classification will occur at the result level rather than once per construct or study.

- A factor measured before and modeled as predicting a later target construct will be classified as a determinant.
- A target construct measured before and modeled as predicting a later outcome will be classified as a harm.
- In a cross-sectional model, an association in which a factor is modeled as the predictor and the target construct as the dependent variable will be assigned to the determinants view.
- In a cross-sectional model, an association in which the target construct is modeled as the predictor and another variable as the dependent variable will be assigned to the harms view.
- An undirected cross-sectional association will be stored once, cross-referenced in the determinants and harms views, and counted once.
- Reciprocal or cross-lagged paths will receive separate result identifiers for each reported direction.
- A prespecified post-intervention endpoint will be classified as effectiveness.
- An unintended intervention outcome will be classified as an adverse event.
- Acceptability, adoption, appropriateness, feasibility, fidelity, cost, penetration, and sustainability will remain implementation outcomes.

A result may be displayed in more than one cross-referenced view but will have one count in the underlying dataset. Directed results will have one primary module; a genuinely undirected concurrent result may have a null primary module and approved linked-module views. This structure prevents, for example, a depression-gaming association from being duplicated merely because it is relevant to determinants and harms.

### Multiple reports, secondary evidence, and overlap

Reports arising from the same study will share a study identifier; reports based on the same participant dataset will also share a dataset-family identifier when applicable. When reports contain the same estimate, it will be charted once from the most complete source and linked to companion reports. Companion publications will be retained for unique outcomes, time points, subgroups, or methods.

Primary studies, systematic reviews or meta-analyses, scoping reviews, and contextual guidelines or consensus statements will be stored at separate source levels. Review conclusions will not be pooled, vote-counted, or merged with primary-study results. A primary study cited by an included review will enter the primary dataset only if independently retrieved and eligible under this protocol; eligibility will never be inherited from a review.

A citation matrix will link each included review to the primary studies identified in its reference list. Secondary reviews may suggest a potential gap, but a gap will be reported only after the direct primary-evidence map is checked. Scoping reviews will remain separate from systematic reviews in charting and appraisal.

### Qualitative and mixed-methods synthesis

Qualitative findings will undergo descriptive framework and content analysis. Initial deductive parent codes will correspond to the seven review questions; reviewers may add inductive subcodes when a finding is not adequately represented. The coding unit will be an author-reported finding, linked to its source locator and any supporting participant quotation. Participant quotations and author interpretations will be stored in separate fields.

Two reviewers will independently code all eligible qualitative findings. A five-study qualitative calibration set, or all available studies if fewer than five exist, will require at least 80% agreement. If the threshold is not met, the codebook will be revised and tested on five new studies, or all remaining new studies if fewer than five exist. Codebook versions, consensus decisions, and retrospective recoding will be documented.

Contradictory, disconfirming, and context-specific findings will be retained. Qualitative findings will be presented beside relevant quantitative mappings but will not be meta-aggregated or treated as pooled evidence. The review will not claim thematic saturation, interpret frequency as importance, or apply GRADE-CERQual.

### Targeted critical appraisal

Two reviewers will independently assign and apply exactly one source-level appraisal method from Table 4, using the current JBI tool suite where specified [63], with disagreements resolved by consensus or third-reviewer adjudication. Method assignment first uses source level; primary empirical sources are then classified by their stated primary aim and the design supporting the primary result. Explicitly integrated mixed-methods sources use MMAT. A multipurpose source receives one method and is not appraised repeatedly merely because it reports secondary analyses or outcomes. A source whose design remains unresolved during screening may be recorded as pending classification, but it must be assigned a listed method before appraisal. If an eligible source cannot be mapped, appraisal pauses and a prospective protocol amendment must add an appropriate method before that source or any later source is appraised; no ad hoc closest-tool substitution is permitted. Appraisal is intended to describe methodological limitations; it will not determine eligibility, generate composite scores, weight findings, rank instruments, or support certainty-of-evidence grades. Results will be reported by checklist item or domain.

**Table 4:** Source-level, non-exclusionary appraisal map.

Evidence design or source level	Appraisal method	Unit	Fields or context charted	Interpretive use
Randomized trial	JBI randomized controlled trial checklist	Source	Randomization, allocation, follow-up, outcome measurement, and analysis	Descriptive context only
Quasi-experimental or single-case intervention	JBI quasi-experimental checklist	Source	Design, confounding, follow-up, outcome measurement, and analysis	Descriptive context only
Cohort or longitudinal observational study	JBI cohort checklist	Source	Selection, exposure and outcome measurement, confounding, and follow-up	Contextualize temporal and adjusted associations
Case-control study	JBI case-control checklist	Source	Selection, exposure measurement, comparability, and confounding	Contextualize causal limitations
Analytical cross-sectional or ecological association study	JBI analytical cross-sectional checklist	Source	Sampling, exposure and outcome measurement, confounding, and analysis	Contextualize concurrent associations
Prevalence or descriptive frequency study	JBI prevalence checklist	Source	Sampling, denominator, case definition, and measurement	Contextualize estimate limitations
Diagnostic-accuracy study	JBI diagnostic test accuracy checklist	Source	Participant spectrum, index test, reference standard, flow, and analysis	Contextualize classification accuracy
Case report or case series	Corresponding JBI case report or case series checklist	Source	Selection, presentation, ascertainment, intervention or exposure, and follow-up	Contextualize descriptive evidence
Economic evaluation	JBI economic evaluation checklist	Source	Alternatives, costs, outcomes, discounting, sensitivity, and incremental analysis	Contextualize economic claims
Qualitative study	JBI qualitative checklist	Source	Congruity, reflexivity, representation, and interpretation	Contextualize development of findings
Explicitly integrated mixed-methods study	MMAT [64]	Source	Qualitative and quantitative components and their integration	Describe component and integration limitations
Psychometric study whose primary aim is measurement-property evaluation	COSMIN Risk of Bias standards [39]	Source	Property-specific design, sample, measurement, and analysis	Contextualize property-specific methods
Intervention systematic review	AMSTAR 2 [65]	Source	Protocol, search, risk of bias, synthesis, and interpretation	Describe limitations separately from primary evidence
Other systematic review	JBI systematic review checklist	Source	Question, search, selection, appraisal, synthesis, and interpretation	Describe limitations separately from primary evidence
Scoping review	PRISMA-ScR reporting fields only [35]	Source	Question, search, selection, charting, and overlap procedures	Reporting fields only; no quality score
Official classification, guideline, or consensus statement	Corresponding JBI Textual Evidence checklist: Expert Opinion, Narrative, or Policy	Source	Issuing body, scope, evidence basis, development methods, and conflicts	Context only; no evidence weighting

Table 4 note: The modified Hoy tool [66] will not be applied because the JBI prevalence checklist is the single prespecified method for prevalence sources. Each included source receives exactly one listed method; multipurpose reports are classified by source level, primary aim, and primary design, not separately by every reported result. No appraisal result will determine eligibility, be converted to a composite score, weight the synthesis, or generate a recommendation hierarchy.

### Contacting source authors

Source authors will be contacted only when missing information concerns an eligibility-critical age subgroup, the identity of the target construct or instrument and cutoff, or a primary estimate essential to a prespecified module output. Routine unreported fields will be coded NR without contact.

One request will be sent to the corresponding author, followed by one reminder after four weeks if necessary [67]. Charting will continue while a response is pending. Contacts, replies, corrections, and unresolved fields will be logged in the associated OSF project; a nonresponse will not cause exclusion. Public logs will omit personal contact information.

### Synthesis and presentation

Synthesis will be descriptive and organized by review question, construct, age-based evidence classification, measurement tier, and source level. No meta-analysis is planned. Numerical findings will be reported as provided, with uncertainty measures when available, and will not be combined across incompatible definitions, instruments, thresholds, or age strata.

Definitions will be summarized in a terminology and classification map. Measurement findings will be presented in an instrument-by-property matrix that preserves language, population, tier, and appraisal. Epidemiological findings will be separated by ascertainment method, instrument, cutoff, tier, and age stratum. Determinants and harms will be mapped by temporality and result role. Intervention findings will separate effectiveness, adverse events, and implementation. Equity reporting will be mapped across PROGRESS-Plus and SAGER fields.

Qualitative findings will be integrated through side-by-side tables and narrative comparison. Secondary reviews will have separate summary tables. The evidence-gap map will identify combinations of constructs, populations, settings, methods, and outcomes for which eligible evidence is sparse or absent; because of language and retrieval boundaries, absence will be described as absence from eligible evidence rather than proof that no evidence exists. Planned outputs are summarized in Table 5.

**Table 5:** Planned presentation outputs.

Output	Primary module	Content	Separation rule
Terminology and classification map	Definitions and classification	Construct labels, nosological status, defining features, impairment, and duration requirements	Diagnoses and problematic-use constructs displayed separately
Measurement matrix	Measurement	Instrument by property by language or population by tier	Frequency of use is not interpreted as quality
Epidemiological matrices	Epidemiology	Estimate, denominator, instrument, cutoff, tier, age, region, and sex or gender	Diagnostic and screen-positive prevalence separated; no pooling
Determinant matrix	Determinants	Factor, I-PACE category, timing, model role, and adjusted estimate	Prospective, concurrent-directed, and undirected results distinguished
Harms table	Harms	Outcome domain, timing, direction, estimate, and adjustment	Prospective and concurrent evidence distinguished
Intervention matrix	Interventions and implementation	TIDieR characteristics, effectiveness, adverse events, and Proctor outcomes	Effectiveness, safety, and implementation separated
Equity table	Equity	PROGRESS-Plus and SAGER reporting and subgroup analyses	Absence of reporting is not treated as absence of inequity
Secondary-evidence tables	Cross-module	Review characteristics and conclusions	Separate from primary evidence and linked through the citation matrix
PRISMA flow diagram	Selection workflow	Search, deduplication, screening, retrieval, exclusions, and inclusion	No date-based exclusion category
Evidence-gap map	Cross-module	Construct by population by setting by method by outcome availability	Describes eligible evidence only; no certainty grade

### Data management and availability

Versioned search files, deduplication records, screening decisions, exclusion reasons, codebooks, translation logs, charting tables, appraisal records, links between included reviews and cited primary studies, and analytic scripts will be maintained in the associated OSF project. Personally identifying correspondence data will not be made public; any public contact ledger will contain only non-identifying process fields.

### Use of artificial intelligence tools

OpenAI Codex (OpenAI; accessed July 2026) assisted with language editing, cross-file consistency checks, reference-metadata checking. Human authors checked and approved all affected text, references, tables, and supporting files. Codex did not make eligibility, appraisal, synthesis, or interpretive decisions and did not generate study data. The authors retain responsibility for accuracy, attribution, interpretations, limitations, and conclusions.

### Ethics and safety

The review will use published or publicly accessible documentary evidence and will not recruit participants, administer interventions, or access individual-level confidential data. Ethics approval, informed consent, sample-size calculation, randomization, blinding, participant safety monitoring, and qualitative saturation are therefore not applicable. The review will preserve accurate attribution and will not reproduce identifying participant quotations beyond what source authors published.

### Study status and timeline

As of July 13, 2026, the search strategy is drafted, PRESS review is pending, and no formal database search, screening, charting, appraisal, or synthesis has begun. PRESS review is projected for July-August 2026, search execution for September 2026, screening and charting through April 2027, and completion of synthesis and domain-focused reports by July 2027. These dates are projections and will be updated transparently if the schedule changes.

## Discussion

### Appropriateness and expected contribution

A scoping review is suited to these questions because it can map divergent terminology, measurement practices, study designs, outcomes, and populations without forcing them into one pooled estimate [31–34]. The review will not determine that all included constructs are equivalent disorders. Instead, it will preserve their nosological differences and show how each has been defined, measured, and studied. This mapping can identify focused questions for later systematic reviews without presenting descriptive breadth as certainty about effects.

The coordinated modules may show where apparent differences arise from measurement choice rather than population differences, where temporal evidence supports or fails to support directional interpretation, and where intervention evidence lacks implementation or equity information. These are anticipated uses of the map, not protocol-stage findings.

### Strengths and limitations

Strengths include one shared sensitive search, explicit age and per-use measurement-tier rules, complete module-specific charting forms, result-level direction and overlap controls, dual human screening and charting, source-language verification, separation of primary and secondary evidence, and a non-exclusionary source-level appraisal method. The staged module structure is intended to make the broad scope operational while retaining one transparent selection pipeline.

The breadth of seven questions may still limit detail within individual modules, and it creates a substantial charting burden. Calibration, assignment to relevant modules, restricted author contact, and versioned guidance reduce but do not eliminate this risk. Heterogeneous definitions, cutoffs, and measurement properties will restrict comparison; the review will not pool estimates or recommend instruments solely from frequency of use.

Language eligibility is limited to English, Spanish, French, German, Portuguese, Arabic, and Hebrew. Source-language verification should reduce translation error within those languages, but exclusion of Chinese, Japanese, Korean, Russian, and other languages may under-represent culturally specific constructs, instruments, determinants, interventions, and policy contexts. Meta-epidemiological findings about the effect of English-language restrictions on pooled estimates [68–70] cannot establish conceptual neutrality in this evolving field. Language exclusions will be counted, and affected outputs will be framed as maps of eligible evidence rather than complete representations of every regional literature.

The strict age policy protects age-specific interpretation but will reclassify some commonly reported samples spanning ages 18–29, or broader ranges, as contextual evidence. Critical appraisal will describe limitations without excluding or weighting studies, so mapped availability should not be interpreted as proof of evidential strength. Automated exact-duplicate detection may introduce error, although human confirmation of non-exact clusters and retained audit logs provide safeguards.

### Dissemination and practical implications

Findings may be reported in two domain-focused manuscripts: definitions and classification, measurement, epidemiology, and determinants (Questions 1–4); and harms, interventions, implementation, and equity (Questions 5–7). Both will derive from the same search and selection process, identify distinct result sets, and cross-reference shared methods without duplicating result counts.

For practice, the measurement map may inform selection of instruments appropriate to a construct, language, and population without substituting for clinical judgment. For service and policy planning, instrument-specific epidemiological and equity maps may clarify where available estimates are comparable. They may also show where surveillance gaps remain. For researchers, the terminology, directionality, intervention, implementation, and gap maps may identify questions suitable for focused systematic reviews or new primary studies. Outputs will be deposited openly in the associated OSF project and shared through peer-reviewed publications and relevant clinical, public-health, education, and youth-health forums.

### Protocol amendments

Material departures from the registration will be dated, justified, and recorded in S3 Appendix. Working materials were deposited in RAV8M, which will also hold future review data and code. Any subsequent substantive departure will receive a dated amendment and, where applicable, a further formal registration update.

## Supporting information

S1 Checklist Completed PRISMA-P checklist with section locators and transparent author-only fields. (DOCX)

S1 Appendix Draft MEDLINE strategy; planned database, Google Scholar, and citation searches; PRESS status; and the July 12, 2026 registry log. (DOCX)

S2 Appendix Complete core and seven module charting guidance forms with critical fields and hypothetical examples. (DOCX)

S3 Appendix Protocol amendment log, implementation status, and projected study timeline. (DOCX)
