# Supplementary material for: Digital behavioral addictions in adolescents and young adults: Protocol for a scoping review of definitions, measurement, epidemiology, determinants, harms, interventions, and equity
Source: PLoS One. 2026 Sep 11;21(9):e0348860. doi: 10.1371/journal.pone.0348860 (PMC13567778; doi:10.1371/journal.pone.0348860)
Supplement: Supplementary file 1 [file pone.0348860.s001.docx]

# S1 Checklist. PRISMA-P 2015 Checklist

| **Section and topic** | **Item** | **How addressed** | **Location or status** |
| --- | --- | --- | --- |
| Title: identification | 1a | Identifies the article as a protocol for a scoping review | Title page |
| Title: update | 1b | Not an update of a completed review | Not applicable |
| Registration | 2 | Identifies OSF Registries and DOI 10.17605/OSF.IO/NPWTV; distinguishes associated project RAV8M | Abstract; Study design, reporting, and registration; Data Availability Statement |
| Authors: contact | 3a | Names, institutional affiliations, corresponding-author email, and available ORCIDs are on the title page. All-author email data and the corresponding physical mailing address must be confirmed in Editorial Manager by the author before upload; they were not available in the locked source package and are not invented here. | Title page; author-only confirmation before upload |
| Authors: contributions and guarantor | 3b | CRediT contributions are reported. Sasha R. Sioni has accepted responsibility as review guarantor. | Author contributions |
| Amendments | 4 | Material departures are dated, compared with the registered or prior position, justified, and assigned an implementation status | Protocol amendments; S4 Appendix |
| Support: sources | 5a | Proposed PLOS field text: “The authors received no specific funding for this work.” Author verification and Editorial Manager entry remain pending; PLOS requires this disclosure outside the manuscript | PLOS Editorial Manager Financial Disclosure field; author confirmation pending |
| Support: sponsor | 5b | Conditional on author confirmation of item 5a, there is no funder or sponsor | Author verification pending; Editorial Manager Financial Disclosure field |
| Support: role | 5c | Conditional on author confirmation of items 5a–5b, there is no funder or sponsor role | Author verification pending; Editorial Manager Financial Disclosure field |
| Rationale | 6 | Explains diagnostic and problematic-use distinctions, fragmented construct-specific evidence, measurement and directional ambiguity, age scope, related protocols, and the need for an integrated map | Introduction |
| Objectives | 7 | Seven linked review questions are stated and mapped to the PCC eligibility framework | Objectives and analytic orientation; Table 2; Eligibility criteria |
| Eligibility criteria | 8 | Defines core and secondary ages, mixed-age contextual use, target constructs, measurement tiers, settings, dates, languages, source types, and conference-material rules | Eligibility criteria; Table 3 |
| Information sources | 9 | Lists nine databases, Google Scholar, websites and organizations, citation searching, and planned registry updates; coverage is from inception | Information sources and search strategy; S2 Appendix |
| Search strategy | 10 | Supplies a reproducible draft MEDLINE via Ovid strategy without date, language, or mandatory domain limits; PRESS is pending | S2 Appendix |
| Study records: data management | 11a | Covidence manages records; automated exact-identifier deduplication is logged; non-exact clusters are human-confirmed; versioned materials are stored in RAV8M | Record management and study selection; Data management and availability |
| Study records: selection | 11b | Two independent reviewers at title or abstract and full text; 50-record and ten-report calibrations; third-reviewer adjudication; one primary full-text exclusion reason | Record management and study selection |
| Study records: data collection | 11c | Pollock dual-table forms; complete core and seven module forms; dual charting; at least five study-module pairs per module and ten unique reports; ≥90% overall and ≥80% within every module and every estimable critical field; exact numeric and source-locator audit; restricted author contact | Data-charting forms and fields; Charting calibration and full charting; Contacting source authors; S3 Appendix |
| Data items | 12 | Lists source, study, sample, result, translation, overlap, and all module-specific variables and rules | Data-charting forms and fields; S3 Appendix |
| Outcomes and prioritization | 13 | No clinical primary outcome is prioritized because this is a descriptive scoping review. Outcomes are prespecified by role: determinants, concurrent associations, harms, effectiveness, adverse events, implementation, qualitative findings, and equity. The seven review questions are mapped without a hierarchy. | Objectives and analytic orientation; Result-role classification; Table 2; S3 Appendix |
| Risk of bias in individual studies | 14 | Assigns exactly one prespecified, non-exclusionary source-level appraisal method by source level, primary aim, and primary design; unresolved classification pauses appraisal until a prospective amendment; no composite score, weighting, exclusion, certainty grade, or recommendation hierarchy | Targeted critical appraisal; Table 4 |
| Synthesis: criteria for quantitative synthesis | 15a | No meta-analysis or quantitative pooling is planned because constructs, designs, instruments, thresholds, and age strata are heterogeneous and the objective is evidence mapping | Objectives and analytic orientation; Synthesis and presentation |
| Synthesis: summary measures and combination | 15b | Not applicable; source estimates and uncertainty are mapped without pooling | Synthesis and presentation |
| Synthesis: additional analyses | 15c | Prespecified stratification by construct, measure tier, instrument, threshold, core versus secondary age, geography, sex or gender, and equity; result-level direction and overlap rules | Module-specific charting; Result-role classification; Table 5 |
| Synthesis: narrative summary | 15d | Descriptive evidence mapping, qualitative side-by-side integration, separate secondary-evidence tables, a citation matrix linking included reviews to cited primary studies, and equity and cross-domain gap maps | Qualitative and mixed-methods synthesis; Synthesis and presentation; Table 5 |
| Meta-biases | 16 | Formal publication-bias tests are not planned. Search, language, registry-indexing, and retrieval limits will be described, and absence in the map will not be treated as proof of absence | Information sources and search strategy; Strengths and limitations; S2 Appendix |
| Confidence in cumulative evidence | 17 | GRADE or another certainty rating is not planned. Source-level appraisal is descriptive and non-exclusionary | Targeted critical appraisal; Synthesis and presentation |

PRISMA-P citation: Shamseer L, Moher D, Clarke M, et al. Preferred reporting items for systematic review and meta-analysis protocols (PRISMA-P) 2015: elaboration and explanation. BMJ. 2015;349:g7647. doi:10.1136/bmj.i4086.
