# Supplementary material for: Digital behavioral addictions in adolescents and young adults: Protocol for a scoping review of definitions, measurement, epidemiology, determinants, harms, interventions, and equity
Source: PLoS One. 2026 Sep 11;21(9):e0348860. doi: 10.1371/journal.pone.0348860 (PMC13567778; doi:10.1371/journal.pone.0348860)
Supplement: Supplementary file 2 [file pone.0348860.s002.docx]

# S2 Appendix. Draft Search Strategy and Registry-Search Log

## Draft MEDLINE strategy via Ovid

The strategy below is a reproducible draft. Subject headings, adjacency, truncation, and field codes will be checked during PRESS review and translated for each platform.

1. Internet Addiction Disorder/

2. Behavior, Addictive/

3. exp Video Games/ or Social Media/ or Smartphone/ or Cell Phone/

4. 2 and 3

5. exp Video Games/

6. (gaming disorder* or internet gaming disorder* or IGD or disordered gaming or problematic gaming or problem gaming or gaming addict* or gaming dependen* or gaming overus* or gaming dysregulat* or maladaptive gaming or pathological gaming).ti,ab,kf.

7. ((video game* or videogame* or video gaming or online game* or internet game*) adj3 (addict* or dependen* or disorder* or dysregulat* or maladapt* or overus* or patholog* or problem* or compuls* or excessive)).ti,ab,kf.

8. ((social media or social network* or social networking site* or Facebook or Instagram or TikTok or Snapchat or Twitter or YouTube) adj3 (addict* or dependen* or disorder* or dysregulat* or maladapt* or overus* or patholog* or problem* or compuls* or excessive)).ti,ab,kf.

9. ((smartphone* or smart phone* or mobile phone* or cellular phone* or cell phone*) adj3 (addict* or dependen* or disorder* or dysregulat* or maladapt* or overus* or patholog* or problem* or compuls* or excessive)).ti,ab,kf.

10. (IGDS9-SF or IGD-20 or "Internet Gaming Disorder Scale" or "Gaming Disorder Test" or GADIT or BSMAS or "Bergen Social Media Addiction Scale" or BFAS or "Bergen Facebook Addiction Scale" or SMDS or "Social Media Disorder Scale" or SAS-SV or "Smartphone Addiction Scale").ti,ab,kf.

11. ((IAT or "Internet Addiction Test" or CIUS or "Compulsive Internet Use Scale") adj5 (gaming or video game* or social media or social network* or smartphone* or mobile phone*)).ti,ab,kf.

12. 1 or 4 or 5 or 6 or 7 or 8 or 9 or 10 or 11

13. exp Child/ or exp Adolescent/ or Young Adult/ or Adult/

14. (child* or preadolescen* or pre-adolescen* or tween* or adolescen* or teen* or youth* or "young people" or "young person*" or adult* or "young adult*" or "emerging adult*" or "transition-age*" or "transition aged").ti,ab,kf.

15. (((elementary or primary or middle or secondary or high) adj2 school*) or student* or undergraduate* or college* or universit*).ti,ab,kf.

16. or/13-15

17. 12 and 16

No date, language, publication-status, or domain limit will be added. Human screening will exclude undifferentiated general Internet or screen use unless the record identifies a named target behavior with separately extractable findings. Generic IAT or CIUS terms are therefore coupled to named target behaviors in line 11 rather than searched alone.

During PRESS review, recall will be challenged against a sentinel set containing at least two eligible records from each target-construct family and at least one record from each age band (10-12, 13-17, 18-24, and 25-29), including a nonstudent community sample in the 25-29 stratum. Before any PRESS testing, the record identifiers and eligibility rationale will be frozen and archived with the PRESS materials in the associated OSF project. Every sentinel record must be retrieved by MEDLINE line 17; a miss will trigger revision of controlled vocabulary or free-text terms and repeat testing before approval. The un-exploded Adult/ heading and adult* text term intentionally over-retrieve some people older than 29 to protect recall for separately extractable 25-29 evidence; age eligibility will be enforced during screening, not by a database limit.

## Database and supplementary-source adaptations

| **Source** | **Planned adaptation and coverage** |
| --- | --- |
| MEDLINE (Ovid) | Draft above; inception to search date; controlled vocabulary plus title, abstract, and keyword fields |
| PsycINFO (Ovid) | Map subject headings and retain proximity and title or abstract fields; inception to search date |
| CINAHL (EBSCO) | Map CINAHL Headings; translate adjacency to N3; inception to search date |
| Scopus | Translate to TITLE-ABS-KEY and W/3; inception to search date |
| Web of Science Core Collection | Translate to Topic (TS=) and NEAR/3; inception to search date |
| Cochrane Library | Translate to MeSH descriptors and title, abstract, keyword fields; inception to search date |
| IEEE Xplore | Translate to metadata and abstract fields; retain computing and engineering terminology; inception to search date |
| ProQuest Dissertations & Theses Global | Translate to title, abstract, and subject fields; inception to search date |
| WHO Global Index Medicus | Translate to platform-supported Boolean and field syntax; inception to search date |
| Google Scholar | Three prespecified supplementary queries; first 200 results per query in relevance order |
| Websites and organizations | Closed six-organization protocol below; exact site-restricted queries, 50-result stopping rule, no recursive crawling, and complete audit log |
| Citation searching | Backward reference-list and forward cited-reference searching for every included source; record platform and date |

ProQuest Dissertations & Theses Global and WHO Global Index Medicus are databases in Fig 1. Google Scholar, websites and organizations, and citation searching are other methods.

## Google Scholar supplementary-search protocol

The following queries will be entered exactly as shown, subject only to a documented correction after PRESS review:

1. ("gaming disorder" OR "internet gaming disorder" OR "problematic gaming") (child OR children OR preadolescent OR preadolescents OR adolescent OR adolescents OR youth OR "young adult" OR "young adults")

2. ("problematic social media use" OR "social media addiction" OR "social media disorder") (child OR children OR preadolescent OR preadolescents OR adolescent OR adolescents OR youth OR "young adult" OR "young adults")

3. ("problematic smartphone use" OR "smartphone addiction") (child OR children OR preadolescent OR preadolescents OR adolescent OR adolescents OR youth OR "young adult" OR "young adults")

For each query, reviewers will screen the first 200 results displayed in relevance order, the lower bound of the 200-300 result range recommended by Haddaway et al. Searches will be supplementary rather than replacements for database or organizational searches. Each search log will record the exact query, date and time, displayed result count, browser and session conditions, signed-in status, number screened, and all retained and unique records. The search will use a logged-out incognito session to reduce account-history effects; no claim will be made that this eliminates personalization or ranking opacity. A contemporaneous PDF, screenshot series, or equivalent audit capture will preserve the rank cutoff.

Haddaway NR, Collins AM, Coughlin D, Kirk S. The role of Google Scholar in evidence reviews and its applicability to grey literature searching. PLoS One. 2015;10(9):e0138237. doi:10.1371/journal.pone.0138237.

## Targeted organization and website protocol

The formal organizational pathway is limited to the following six bodies and listed domains. The selection covers the two governing diagnostic systems, international child/youth and digital-policy bodies, and a major clinical-guideline producer. It is supplementary and is not represented as an exhaustive search of all agencies or countries.

| **Organization** | **Prespecified domain(s)** | **Role in the targeted search** |
| --- | --- | --- |
| World Health Organization and ICD | who.int; icd.who.int | ICD classification, public-health guidance, and official explanatory materials |
| American Psychiatric Association | psychiatry.org; psychiatryonline.org | DSM classification and official psychiatric guidance |
| UNICEF | unicef.org | Child and adolescent policy, rights, and digital-wellbeing guidance |
| UNESCO | unesco.org | Education, youth, and digital-policy guidance |
| International Telecommunication Union | itu.int | International digital-technology and child/youth policy guidance |
| National Institute for Health and Care Excellence | nice.org.uk | Clinical and service-delivery guidance |

For every listed domain, reviewers will run each of these exact logged-out, site-restricted Google Web Search queries in an incognito session: site:DOMAIN "gaming disorder"; site:DOMAIN "internet gaming disorder"; site:DOMAIN "problematic social media use"; site:DOMAIN "social media addiction"; site:DOMAIN "problematic smartphone use"; and site:DOMAIN "smartphone addiction". All displayed results will be screened when 50 or fewer are returned; otherwise, the first 50 results in relevance order will be screened. No date filter will be applied. Reviewers will screen the landing result and any directly attached document but will not recursively crawl further links. An included organizational source must be an official classification, guideline, or consensus statement that addresses a named target construct or clarifies its boundary; news, advocacy, ad hoc surveillance, and standalone policy or implementation documents may serve only as citation leads and will not enter the formal evidence map. Non-English documents discovered through this pathway remain eligible under the seven-language rule and require the same source-language human verification.

For each query, the log will retain organization, domain, exact query, date and time, displayed count, browser/session and signed-in status, rank screened, title, URL, document date/version, decision, exclusion reason, and any directly attached file. A PDF or screenshot series will preserve the result order and cutoff. If a listed domain is unavailable, the attempt and error will be logged; no unlisted substitute organization or domain may be added without a dated protocol amendment. The English-phrase site queries interrogate the organizations' principal indexed web presence; multilingual retrieval is expected primarily from the unrestricted database searches and citation searching, so the organization pathway will not be described as language-comprehensive.

## Citation searching

Backward reference-list searching and forward cited-reference searching will be conducted for all included primary, secondary, and contextual sources. Reviewers will record the seed source, search direction, platform, date, number screened, records retained, and links to resulting source identifiers. Citation searching follows TARCiS terminology. The original TARCiS article and its published correction are both cited in the main manuscript.

## Deduplication and automation boundary

Exact-identifier duplicates may be collapsed automatically in Covidence, with an audit record retained. Non-exact and near-duplicate clusters require human confirmation; ambiguous clusters require a second human check. Automation may not exclude a record for eligibility or make a full-text exclusion decision.

## Language retrieval and verification

No database language filter will be applied. English, Spanish, French, German, Portuguese, Arabic, and Hebrew records are eligible. Every final non-English title-and-abstract decision, full-text decision, exclusion reason, and extracted finding will be checked against the original by a source-language-proficient human. Machine translation may prepare a working rendering but cannot determine eligibility or a charted value.

## Date-bounded registry and comparator search

### Scope

The comparator target was an independent protocol combining the same four eligible target construct families (ICD-11 Gaming Disorder, DSM-5-TR Internet Gaming Disorder, problematic social media use, and problematic smartphone use), seven evidence domains (definitions or classification, measurement, epidemiology, determinants, harms, interventions or implementation, and equity), and ages 10-29. Searches were conducted on 12 July 2026. Undifferentiated Internet-overuse constructs were not treated as equivalent comparators. The authors' own NPWTV, UYBA2, and Q7AUZ records were not treated as independent comparators.

### OSF Registries

Interface: OSF Registries API title filter, https://api.osf.io/v2/registrations/?filter[title]=QUERY&page[size]=100; pagination continued until links.next was null.

| **Exact title-filter query** | **Hits** | **Closest records and interpretation** |
| --- | --- | --- |
| digital behavioral addiction | 3 | NPWTV, UYBA2, and Q7AUZ are author-associated records, not independent comparators |
| gaming disorder | 94 | Included narrower scoping and intervention protocols such as Y2F94, RXKJU, and TXWBH |
| problematic social media | 24 | Included narrower intervention, determinant, and measurement protocols such as P58B6, NAFM9, and ZXE4B; VBY6P was withdrawn |
| problematic smartphone | 15 | Included narrower reviews such as FJYHA |

Finding: related active records exist. No independent inspected OSF title or description record combined all four construct families, all seven modules, and ages 10-29. The negative scope inference is limited because title filtering may miss different terminology.

### PROSPERO

Interface: PROSPERO public search API used by https://www.crd.york.ac.uk/PROSPERO/search; endpoint POST https://www.crd.york.ac.uk/PROSPERO/api/search. Request fields were term, page=1, nperpage, sort, sortorder, filters=[], download=false, and actual equal to the displayed query. The public client supplied prospero-access-token: null, prospero-auth-token: Base64(current Unix-epoch time in milliseconds), and Content-Type: application/json.

| **Exact query** | **Hits** | **Interpretation** |
| --- | --- | --- |
| "digital behavioral addiction" | 2 | Included a measurement-only short-form-video-use record, CRD420261409703 |
| "gaming disorder" AND (adolescent OR youth OR "young adult") | 145 | Numerous narrower prevalence, intervention, and determinant registrations |
| ("problematic social media use" OR "social media addiction") AND (adolescent OR youth OR "young adult") | 102 | Numerous narrower intervention and determinant registrations |
| ("problematic smartphone use" OR "smartphone addiction") AND (adolescent OR youth OR "young adult") | 115 | Numerous narrower prevalence and intervention registrations |

Critical limitation: PROSPERO does not register scoping reviews. Its results demonstrate adjacent systematic-review activity but cannot establish the absence of a scoping-review protocol.

Selected directly inspected records: https://www.crd.york.ac.uk/PROSPERO/view/CRD420261409703; https://www.crd.york.ac.uk/PROSPERO/view/CRD42021282478; https://www.crd.york.ac.uk/PROSPERO/view/CRD42023399423; https://www.crd.york.ac.uk/PROSPERO/view/CRD420261389179; https://www.crd.york.ac.uk/PROSPERO/view/CRD420261421899; and https://www.crd.york.ac.uk/PROSPERO/view/CRD42023448855.

### INPLASY

Interface: the standard WordPress search form, GET https://inplasy.com/?s=QUERY. Counts are archive-page hits, not unique protocols, because terms may occur in multiple records on one archive page.

| **Exact query** | **Archive-page hits** |
| --- | --- |
| digital behavioral addiction | 2 |
| digital addiction adolescent | 4 |
| gaming disorder adolescent | 2 |
| social media addiction adolescent | 3 |
| smartphone addiction adolescent | 0 |
| internet addiction young adult | 3 |

Direct follow-up verified related but narrower records, including INPLASY202470023 (ostracism and digital addiction), INPLASY2024110114 (Internet-addiction prevalence in university students), and INPLASY2025100105 (sports interventions for adolescent Internet addiction). INPLASY accepts scoping and mapping reviews, but its interface does not support a high-confidence registry-wide negative claim.

Direct records: https://inplasy.com/inplasy-2024-7-0023/; https://inplasy.com/inplasy-2024-11-0114/; and https://inplasy.com/inplasy-2025-10-0105/.

### JBI Evidence Synthesis

JBI Evidence Synthesis is a journal corpus, not a prospective registry. Its public search endpoint was POST https://www.ovid.com/publicapi/content-service/search/ovid/journals/jbisrir/results, with queryString, filters pdt=all and ct=article, page=1, and pageSize=100. The API returned noisy phrase matches: digital behavioral addiction 10; gaming disorder 10; problematic social media use 38; social media addiction 16; problematic smartphone use 11; smartphone addiction 8; and internet addiction 24. The closest directly verified published protocol was Ashok et al., *Association of nomophobia and health consequences among university students: a systematic review protocol* (https://doi.org/10.11124/JBIES-25-00252).
