# Supplementary material for: Digital behavioral addictions in adolescents and young adults: Protocol for a scoping review of definitions, measurement, epidemiology, determinants, harms, interventions, and equity
Source: PLoS One. 2026 Sep 11;21(9):e0348860. doi: 10.1371/journal.pone.0348860 (PMC13567778; doi:10.1371/journal.pone.0348860)
Supplement: Supplementary file 3 [file pone.0348860.s003.docx]

# S3 Appendix. Complete Data-Charting Guidance Forms

## Purpose and use

This appendix contains the complete initial Table A charting guidance, which will be version-locked before calibration. Table B will be the populated dataset. The forms cover the shared core and all seven modules: definitions and classification, measurement, epidemiology, determinants, harms, interventions and implementation, and equity. Every worked example is hypothetical and does not represent a finding of this review.

Two reviewers will independently chart at least five study-module pairs per module and at least ten unique reports overall. If fewer than five pairs exist for a module, all available pairs will be used. The denominator includes every jointly applicable structured categorical field. NR, NA, and UNCLEAR are categorical decisions, and any mismatch involving these codes counts as disagreement. A field is excluded only when both reviewers independently select NA and reconciled applicability confirms that it does not apply. The gate is at least 90% agreement overall and at least 80% in every module and every critical field with an applicable comparison. Numerical values and source locators require exact comparison and are audited separately. A critical field with no applicable paired ratings is not estimable, not passed: additional eligible pairs are added when possible; if all available pairs are correctly NA, a third reviewer confirms deferral and the gate reopens when the first applicable source appears. A failing module or critical field triggers a versioned rule revision and five new affected-module pairs, or all remaining pairs if fewer than five exist. All earlier records affected by a rule change will be re-charted.

Allowed missingness codes are NR (not reported), NA (not applicable), and UNCLEAR (reported but not resolvable). Reviewers may not infer a value from absence. Source locators use page, section, table, figure, paragraph, appendix, or other stable locator.

## Core source, study, sample, and result fields

| **Field** | **Definition** | **Allowed values** | **Decision rule and critical status** | **Hypothetical example** |
| --- | --- | --- | --- | --- |
| source_id | Unique record for every retrieved document | Stable alphanumeric | Never reused; critical | SRC000145 |
| report_id | Unique report or publication | Stable alphanumeric | One per report; critical | RPT000088 |
| study_id | Underlying primary-study identity | Stable alphanumeric, NA, or UNCLEAR | Assign to primary research; link companion reports only with direct evidence; critical | STU000052 |
| sample_id | Distinct analyzed primary-study sample or wave | Stable alphanumeric or NA | Assign when an analyzed sample exists; separate samples, cohorts, or waves; critical | SAM000052A |
| dataset_family_id | Family of reports using the same participant dataset | Stable alphanumeric, NA, UNCLEAR | Do not merge only because country or authors overlap | DAT000019 |
| review_id | Identifier for secondary evidence | Stable alphanumeric or NA | Used only for systematic, meta-analytic, or scoping reviews | REV000013 |

### Core fields (continued)

| **Field** | **Definition** | **Allowed values** | **Decision rule and critical status** | **Hypothetical example** |
| --- | --- | --- | --- | --- |
| result_id | Unique quantitative or qualitative finding | Stable alphanumeric | One direction, estimate, or author-reported finding per row; critical | RES000912 |
| Citation | Full bibliographic identity | Text and identifiers | Verify DOI, PMID, year, and version | Hypothetical Author, 2024 |
| source_level | Evidence level | primary, systematic_review, scoping_review, guideline_consensus | guideline_consensus includes eligible official classifications; do not merge levels; critical | primary |
| Design | Design reported or verified | RCT, quasi-experimental or single-case intervention, cohort or longitudinal observational, case-control, analytical cross-sectional or ecological association, prevalence or descriptive frequency, diagnostic accuracy, case report, case series, economic evaluation, qualitative, mixed methods, psychometric, systematic review, scoping review, official classification, guideline or consensus, other_pending | Use methods, not title alone; other_pending must be resolved before appraisal; critical | cohort |
| appraisal_method | Exactly one source-level appraisal method | One method listed in manuscript Table 4 | Assign by source level, then stated primary aim and primary design; multipurpose sources receive one method; unresolved classification pauses appraisal and requires a prospective amendment; critical | JBI cohort checklist |
| Country and region | Study location | Country; World Bank group; WHO region | Record each country in multisite work | Hypothetical country; upper-middle income |
| Setting | Recruitment or delivery setting | Clinical, school, university, community, household, online, mixed, other | Distinguish recruitment from intervention setting | school |
| Sample size | Analyzed denominator for the result | Integer plus denominator definition | Do not use enrolled N when result N differs; critical | n=842 complete cases |
| Age evidence | Age range and extractability | Reported statistics plus exact subgroup | Record range, mean, median, and subgroup source | 15-19 subgroup, Table 2 |
| population_evidence | Eligibility role of age data | core_10_24, secondary_25_29, mixed_contextual, exclude | Assign per charted sample or result; one report may carry multiple codes across extractable strata; mean or median alone never establishes core or secondary eligibility; critical | mixed_contextual for inseparable ages 18-29 |

### Core fields (continued)

| **Field** | **Definition** | **Allowed values** | **Decision rule and critical status** | **Hypothetical example** |
| --- | --- | --- | --- | --- |
| Sex and gender | Terms and distributions used by source | Verbatim terms and counts | Do not replace sex with gender or vice versa | self-reported gender: 52% girls |
| Source language | Language of report | English, Spanish, French, German, Portuguese, Arabic, Hebrew, other | Critical for eligibility and translation log | Portuguese |
| Translation provenance | Human and optional machine workflow | Language; tool/version; verifier/proficiency; date; corrections | Every final non-English decision and finding requires source-language human check; critical | DeepL working rendering; verified by fluent reviewer |
| Target construct | Named target behavior or disorder | GD, IGD, PSMU, PSU, other eligible label | Undifferentiated Internet use is not eligible; critical | problematic social media use |
| Instrument use | Instrument, version, language, respondent, administration | Text plus structured fields | Tier is assigned for each use, not globally | BSMAS, Spanish, self-report |
| measure_tier | Evidence tier for this instrument use | Tier 1, Tier 2, Tier 3, N/A | No tier-based exclusion; critical | Tier 2 |
| primary_module | One primary result destination | definitions, measurement, epidemiology, determinants, harms, interventions, equity, null | null only for genuinely undirected concurrent evidence; critical | harms |
| linked_modules | Additional cross-referenced views | Array of module names | Cross-reference without duplicate storage or counting | [determinants, harms] |
| result_role | Direction and analytic role | determinant, determinant_concurrent, harm, harm_concurrent, concurrent_undirected, effectiveness, adverse_event, implementation, qualitative_finding, other | Apply result-level hierarchy; critical | concurrent_undirected |

### Core fields (continued)

| **Field** | **Definition** | **Allowed values** | **Decision rule and critical status** | **Hypothetical example** |
| --- | --- | --- | --- | --- |
| Temporality | Timing between predictor and outcome | prospective, concurrent, retrospective, unclear, NA | Do not call cross-sectional evidence prospective | prospective |
| Directionality | Modeled direction | X-to-DBA, DBA-to-Y, reciprocal, undirected, NA | Reciprocal paths receive separate result IDs | DBA-to-later-depression |
| Estimate and uncertainty | Reported numeric result | Estimate, unit, CI/SE/SD, p value as reported | Transcribe exactly; do not calculate unless prespecified; critical numeric audit | adjusted OR 1.42, 95% CI 1.10-1.82 |
| Adjustment | Confounder handling | Unadjusted; adjusted plus covariates; NA | Record model number and covariates | age, sex, baseline symptoms |
| Source locator | Exact location in report | Page, section, table, figure, row, quotation locator | Required for every charted finding; critical exact audit | Table 3, model 2, row 5 |
| Overlap link | Related result, report, study, or review | Identifier array | Same estimate is charted once and linked | RPT000089; RES000912 |
| Critical-field status | Whether field is in calibration gate | Yes or no | All fields marked critical above are Yes | Yes |

## Module 1: Definitions and classification

| **Field** | **Definition** | **Allowed values** | **Decision rule and critical status** | **Hypothetical example** |
| --- | --- | --- | --- | --- |
| Term used | Source's primary label | Verbatim term plus normalized construct | Preserve source language and translated term; critical | uso problemático de redes sociales / PSMU |
| Nosological status | Status claimed by source | ICD diagnosis, DSM condition for further study, research construct, symptom screen, unclear | Do not upgrade source status | research construct |
| Classification system | Formal system referenced | ICD-11, DSM-5 or DSM-5-TR, none, other | Record edition and locator | ICD-11 |
| Definitional approach | Conceptual basis | Formal diagnostic, component model, behavioral, neurobiological, hybrid, atheoretical, unclear | Code only stated or directly operationalized basis | component model |
| Core features | Defining features | Impaired control, priority or salience, persistence despite harm, functional impairment, tolerance, withdrawal, other | Multi-select and quote locator | impaired control; functional impairment |
| Functional impairment | Whether required | Required, optional, absent, unclear | Distinguish scale item from case requirement; critical | required |
| Duration | Required symptom duration | Exact duration, none, unclear | Record diagnostic threshold separately | 12 months |
| Severity framing | Construct representation | Categorical, dimensional, hybrid, unclear | Dichotomization plus continuous score is hybrid | hybrid |
| Threshold basis | Basis for classification | Diagnostic algorithm, validated cutoff, sample-derived cutoff, author-defined, none | Do not imply clinical diagnosis from a scale cutoff | ROC-derived screen cutoff |
| Definitional citation | Source cited for definition | Full citation or NR | Verify that cited source actually defines construct | WHO ICD-11 page |
| Alignment statement | Relationship to established criteria | Explicit, partial, none, contradictory | Record source's statement, not reviewer preference | partial DSM alignment |
| Historical stratum | Diagnostic or instrument milestone | Year or era label | Chart only; never use for eligibility | pre-ICD-11 formalization |

## Module 2: Measurement

COSMIN v2.0 criteria are applied by property. A single binary validated or unvalidated label is prohibited. Internal consistency is interpretable only when structural validity or unidimensionality is adequate. Structural-validity criteria are alternatives appropriate to the analysis type, not a requirement to meet every fit index. Per-use tier provenance is linked to the separate validation-evidence log; two reviewers verify each tier and retain exact searches and identifiers.

| **Field** | **Definition** | **Allowed values** | **Decision rule and critical status** | **Hypothetical example** |
| --- | --- | --- | --- | --- |
| Instrument identity | Name, acronym, version, item count | Text | Verify original and adaptation versions; critical | Hypothetical Gaming Scale-9 |
| Construct and population | Intended construct and target group | Text plus age and setting | Record mismatch between intended and actual use | adolescent community IGD screen |
| Language and adaptation | Language and adaptation process | Forward/back translation, cognitive interview, pilot, none, unclear | Assign tier from evidence, not translation claim alone; critical | forward/back translation only |
| Per-use evidence tier | Tier 1, 2, 3, or N/A | Structured value | Apply locked tier definitions; critical | Tier 2 |
| Validation source | Identifiable evidence supporting the tier | DOI, PMID, manual URL, developer record, or none identified | Tier 1 or 2 requires a traceable source; critical | doi:10.xxxx/example |
| Validation lookup | Reproducible search provenance | Date, exact query, PubMed/PsycINFO/COSMIN result, citation chain | Retain every source checked and the no-result state; critical | 2026-10-03; instrument + Spanish + validity |
| Validation match | Fit of located evidence to current use | Language match, age-population match, construct/version match | Record each dimension separately; do not infer a full match | language yes; youth no; version yes |
| Tier verification | Independent decision and reconciliation | Reviewer 1 tier, Reviewer 2 tier, final tier, adjudicator if used | Resolve before synthesis; version any change; critical | T2/T2; final T2 |
| Content validity | Relevance, comprehensiveness, comprehensibility | COSMIN result plus evidence details | Record patient and professional involvement and methods | relevance assessed; comprehensiveness NR |
| Structural validity | Dimensional structure | EFA/PCA, CFA, IRT/Rasch indices and result | Use COSMIN v2.0 alternative criteria appropriate to method; critical | CFA CFI 0.96; RMSEA 0.07; sufficient by CFI |
| Internal consistency | Interrelatedness within a unidimensional score | Alpha, omega, other | Rate only when structural validity or unidimensionality is adequate; critical | omega 0.88; interpretable after adequate CFA |

### Module 2: Measurement (continued)

| **Field** | **Definition** | **Allowed values** | **Decision rule and critical status** | **Hypothetical example** |
| --- | --- | --- | --- | --- |
| Cross-cultural validity or invariance | Equivalence across groups or languages | DIF; configural, metric, scalar; other | Record groups, sample sizes, and criteria | scalar invariance across sex groups |
| Reliability | Reproducibility across stable occasions or raters | ICC, kappa, other plus interval | Distinguish reliability from agreement and internal consistency | ICC 0.79 over 2 weeks |
| Measurement error | Absolute error | SEM, SDC, LoA, other | Record units and relation to minimal important change if available | SDC 4.2 points |
| Criterion validity | Agreement with a defensible gold standard | AUC, sensitivity, specificity, correlation | Record the proposed standard and justify whether it is a gold standard | AUC 0.81 versus structured diagnostic interview |
| Construct validity | Prespecified hypothesis testing | Convergent, divergent, known-groups, other | Record hypothesis and result; post hoc direction alone is insufficient | predicted correlation 0.50-0.70, observed 0.62 |
| Responsiveness | Ability to detect change | Effect or hypothesis test | Apply only when change is relevant and study design supports it | change correlated 0.55 with anchor |
| Cutoff derivation | Threshold and derivation method | Diagnostic algorithm, ROC or Youden, percentile, latent class, author-defined, other | Cutoff is not itself a COSMIN property; chart method and reference standard | score 31 by Youden index |
| Interpretability | Meaning assigned to scores | Distribution, floor or ceiling, meaningful change, norms | Do not equate interpretability with validity | 12% ceiling effect |
| Feasibility | Practical burden | Time, cost, licensing, training, accessibility | Record as descriptive | 5 minutes; free for research use |
| Property appraisal | COSMIN Risk of Bias result | Property-specific item ratings | No composite score; report property by property | structural validity: very good |
| Source locator | Location of method and result | Exact page/table/appendix | Required for every property and numeric value; critical | Supplement p. 4, Table S2 |

## Module 3: Epidemiology

| **Field** | **Definition** | **Allowed values** | **Decision rule and critical status** | **Hypothetical example** |
| --- | --- | --- | --- | --- |
| Epidemiologic unit | Population and time frame | Point, period, lifetime, incidence, other | Do not mix time frames | past-12-month screen-positive prevalence |
| Sampling frame | Source population | Probability, census, convenience, clinical, platform, other | Record recruitment and response rate | school census, 73% response |
| Denominator | Participants eligible for estimate | Integer and exclusions | Use estimate-specific denominator; critical | 812 with complete scale data |
| Ascertainment | How cases or screens were defined | Diagnostic interview, algorithm, scale cutoff, author-defined | Critical for terminology | scale cutoff 32 |
| Measure tier | Per-use tier | Tier 1, 2, 3 | Tier 3 never labeled disorder prevalence; critical | Tier 1 |
| Estimate label | Permitted label | Diagnostic prevalence, screen-positive prevalence, symptom frequency, other | Apply locked terminology; critical | screen-positive prevalence |
| Estimate | Numerator, denominator, percent or rate | Exact numeric | Record published precision and CI | 64/812, 7.9%, 95% CI 6.1-9.7 |
| Instrument and cutoff | Instrument version and threshold | Text and numeric | Keep separate in outputs | Hypothetical Scale cutoff ≥32 |
| Age stratum | Eligible age role | 10-14, 15-19, 20-24, 25-29 secondary, mixed contextual | Mixed contextual cannot enter age-specific estimates; critical | 15-19 |
| Sex or gender stratum | Source-defined subgroup | Verbatim term and estimate | Do not convert terminology | girls, 6.2% |
| Geography | Country, region, urbanicity | Structured and verbatim | Preserve multisite differences | urban and rural strata |
| Weighting | Survey weights or design correction | None, weights, cluster correction, unclear | Record weighted and unweighted N | post-stratification weights |
| Risk of bias | JBI prevalence checklist items | Item or domain results | No Hoy duplication, composite score, or exclusion | sampling frame item: yes |
| Multiple estimates | Related cutoffs or instruments | Separate result IDs and links | Do not select one post hoc | cutoff 28 and 32 as separate rows |
| Source locator | Exact location | Page, table, row, supplement | Critical numeric audit | Table 2, row ages 15-19 |

## Module 4: Determinants

| **Field** | **Definition** | **Allowed values** | **Decision rule and critical status** | **Hypothetical example** |
| --- | --- | --- | --- | --- |
| Putative determinant | Factor modeled in relation to target construct | Verbatim and normalized term | Do not call an undirected correlation causal | loneliness |
| I-PACE category | Organizing category | Person, Affect, Cognition, Execution, multiple, not applicable | Coding is organizational, not causal proof | Affect |
| Exposure timing | Timing of factor | Baseline, repeated, concurrent, retrospective, unclear | Critical for result role | baseline |
| Outcome timing | Timing of target construct | Later, concurrent, retrospective, unclear | Critical for result role | 12-month follow-up |
| Result role | Determinant classification | determinant, determinant_concurrent, concurrent_undirected, other | Apply hierarchy; critical | determinant |
| Model direction | Predictor and dependent variable | X-to-DBA, undirected, reciprocal | Cross-lagged directions are separate results | loneliness-to-later-PSMU |
| Association | Estimate and uncertainty | Exact numeric | Record adjusted and unadjusted separately | adjusted β=0.18, 95% CI 0.06-0.30 |
| Covariates | Adjustment set | Verbatim list | Record model number and rationale if stated | age, sex, baseline PSMU |
| Mediator or moderator | Analytic function | Direct, mediator, moderator, moderated mediation, none | Record method and temporality | mediator |
| Design support | Design relevant to inference | Prospective, concurrent, case-control, qualitative, other | Causal wording prohibited for concurrent data | prospective cohort |
| Population role | Age evidence code | Core, secondary, mixed contextual | Mixed contextual cannot support age-specific quantitative determinant output | core_10_24 |
| Primary and linked modules | Storage and views | determinants plus optional links | Undirected correlation stored once with null primary module or approved neutral rule | primary=determinants; linked=harms |
| Source locator | Exact model location | Page, table, model, row | Critical exact audit | Table 4, model 3 |

## Module 5: Harms

| **Field** | **Definition** | **Allowed values** | **Decision rule and critical status** | **Hypothetical example** |
| --- | --- | --- | --- | --- |
| Outcome domain | Type of adverse outcome | Mental or emotional, sleep or circadian, physical, social or family, academic or occupational, neurocognitive, other | Multi-domain findings receive separate result IDs | sleep or circadian |
| Outcome measure | Instrument or record used | Name, version, language | Assign measurement tier where relevant | hypothetical sleep scale |
| Target-construct timing | Timing of DBA measure | Baseline, repeated, concurrent, unclear | Critical for direction | baseline |
| Outcome timing | Timing of harm measure | Later, concurrent, retrospective, unclear | Critical for direction | 6-month follow-up |
| Result role | Harm classification | harm, harm_concurrent, concurrent_undirected, other | Apply hierarchy; critical | harm |
| Model direction | Predictor and dependent variable | DBA-to-Y, undirected, reciprocal | Separate reciprocal paths | IGD-to-later-sleep-problem |
| Association | Estimate and uncertainty | Exact numeric | Do not translate association into causation | adjusted RR 1.30, 95% CI 1.10-1.54 |
| Covariates | Adjustment set | Verbatim list | Record baseline outcome adjustment | age, sex, baseline sleep |
| Adverse-event distinction | Whether intervention-emergent | Observational harm, intervention adverse event, unclear | Intervention adverse events are stored in intervention module | observational harm |
| Population role | Age evidence code | Core, secondary, mixed contextual | Mixed contextual cannot support age-specific quantitative harm output | secondary_25_29 |
| Primary and linked modules | Storage and views | harms plus optional links | One stored result and one count | primary=harms; linked=determinants |
| Source locator | Exact model location | Page, table, model, row | Critical exact audit | Figure X, cross-lagged path B |

## Module 6: Interventions and implementation

| **Field** | **Definition** | **Allowed values** | **Decision rule and critical status** | **Hypothetical example** |
| --- | --- | --- | --- | --- |
| Brief name | Source's intervention name | Text | Do not invent a branded name | Skills for Balanced Gaming |
| Why | Theory or rationale | Text and named framework | Record stated basis | CBT model of gaming cognitions |
| Materials | Physical or digital materials | Text; manualized yes or no | Record availability and access | therapist manual and workbook |
| Procedures | Activities and sequence | Text plus components | Record session-level detail where available | psychoeducation, restructuring, relapse planning |
| Provider | Deliverer and training | Profession, training, supervision | Distinguish automated from clinician-delivered | licensed psychologist, 2-day training |
| How | Delivery mode | Individual, group, family, school, app, hybrid, other | Multi-select with primary mode | group plus app reminders |
| Where | Delivery setting | Clinical, school, community, online, home, mixed | Distinguish study setting from delivery setting | school counseling office |
| When and how much | Dose and schedule | Sessions, frequency, duration, total period | Transcribe planned and actual separately | 8 weekly 60-minute sessions |
| Tailoring | Planned personalization | Yes, no, NR plus description | Do not infer tailoring from flexible delivery | examples tailored to preferred games |
| Modifications | Changes during study | Yes, no, NR plus description | Version and timing required | two sessions moved online |
| Fidelity planned | Planned fidelity assessment | Method and threshold | Separate from achieved fidelity | 20% session audio review |
| Fidelity actual | Delivery achieved | Numeric or qualitative | Record denominator and assessor | 91% checklist adherence |
| Comparator | Comparison condition | Waitlist, usual care, active, none, other | Record content and intensity | attention-control group |
| Allocation and follow-up | Design features | Randomization, concealment, follow-up times | Link to appraisal | individual randomization; 3 months |
| Effectiveness result | Intended outcome | Estimate, CI, time point | One result per outcome and direction | mean difference -2.1, 95% CI -3.5 to -0.7 |
| Adverse event | Unintended outcome | Type, seriousness, relatedness, denominator | Absence of reporting is NR, not zero | NR |
| Acceptability | Perceived agreeableness | Scale, rate, qualitative finding | Preserve perspective and time point | participant satisfaction 4.2/5 |
| Adoption | Uptake by settings or providers | Rate or description | Distinguish participant enrollment | 6/10 schools adopted |
| Appropriateness | Perceived fit | Scale or qualitative finding | Record stakeholder | teachers rated fit as high |
| Feasibility | Practical execution | Rate, barrier, facilitator | Record resource context | scheduling was main barrier |
| Implementation fidelity | Adherence at implementation level | Percentage or rating | Link but do not duplicate TIDieR fidelity | 91% protocol adherence |
| Implementation cost | Cost of delivery or implementation | Currency, year, perspective | Specify what was costed | USD 120 per participant, 2025 |
| Penetration | Reach within service setting | Numerator, denominator, description | Distinguish from trial participation | 18% of eligible students reached |
| Sustainability | Maintenance over time | Time point and status | NR if no post-implementation evidence | maintained at 3/5 sites at 12 months |
| Population role | Age evidence code | Core, secondary, mixed contextual | Mixed contextual may describe implementation only, not effectiveness | mixed_contextual for delivery description |
| Source locator | Exact intervention or result location | Page, table, supplement, protocol | Critical for TIDieR and result values | Appendix 2, session manual |

## Module 7: Equity

| **Field** | **Definition** | **Allowed values** | **Decision rule and critical status** | **Hypothetical example** |
| --- | --- | --- | --- | --- |
| Place of residence | Geography within country | Urban, rural, remote, mixed, NR, other | Record source definition | rural district |
| Race, ethnicity, culture, language | Source-described identity | Verbatim categories | Do not harmonize without a documented crosswalk | source-defined ethnic categories |
| Occupation | Participant or household occupation | Text, category, NR | For minors, caregiver occupation may be separate | caregiver occupation recorded |
| Gender or sex | Source terminology and analysis | Verbatim term, categories, method | Apply SAGER distinction; critical | sex assigned at birth analyzed |
| Religion | Religious identity or context | Verbatim, NR | Do not infer from country | NR |
| Education | Participant or household education | Grade, attainment, institution type | Distinguish individual from caregiver | grade 10 |
| Socioeconomic status | Material or income indicator | Income, deprivation, assets, insurance, other | Record operationalization | household asset index quintile |
| Social capital | Social relationships and resources | Scale, proxy, qualitative finding | Do not equate social support automatically | neighborhood cohesion scale |
| Plus factors | Other equity-relevant characteristics | Age, disability, sexual orientation, migration, care status, other | Record prespecified and emergent factors | sexual orientation reported |
| Representation | Inclusion and exclusion of groups | Eligibility, recruitment, sample distribution | Absence of a group is not proof of exclusion | excluded youth without smartphones |
| Accessibility or adaptation | Accommodations and cultural or linguistic tailoring | Yes, no, NR plus description | Link to intervention or instrument | right-to-left interface adaptation |
| Subgroup analysis | Analysis by equity factor | Descriptive, stratified, interaction, adjusted, inequity analysis | Do not call a descriptive difference an inequity without analysis | sex interaction p=0.03 |
| Reporting gap | Missing equity information | Factor-specific present or absent | Absence of reporting is not absence of inequity | race or ethnicity not reported |
| Source locator | Exact location | Page, table, appendix | Critical for subgroup estimate and terminology | Table 1 and model 4 |

## Qualitative coding and mixed-methods integration

The initial deductive parent codes correspond to the seven review questions. Inductive subcodes may be added when an author-reported finding does not fit the current codebook. The coding unit is an author-reported finding, not a whole article. Each finding receives a result_id, source locator, participant group, context, and a separate field for any supporting participant quotation. Quotations and author interpretations may not be merged.

Two reviewers will independently code every eligible qualitative finding. A five-study qualitative calibration set, or all available studies if fewer than five exist, must reach at least 80% agreement. A failed calibration triggers a revised, versioned codebook and five new studies, or all remaining new studies if fewer than five exist. Rule changes trigger retrospective recoding of affected findings. Negative, contradictory, disconfirming, and context-specific cases are retained. Qualitative findings are integrated beside relevant quantitative maps through side-by-side tables and narrative comparison; they are not meta-aggregated, pooled, treated as saturation evidence, ranked by frequency, or assessed with GRADE-CERQual.

| **Qualitative field** | **Definition** | **Allowed values** | **Decision rule** | **Hypothetical example** |
| --- | --- | --- | --- | --- |
| Parent code | Review-question domain | One of seven modules | One primary code, links permitted | interventions |
| Inductive subcode | Emergent concept | Versioned free text | Add only with definition and example | peer accountability |
| Finding type | Source presentation | Author interpretation, participant quotation, mixed | Store quotations separately | author interpretation |
| Finding text | Verbatim or faithful verified rendering | Text | Preserve source language and verified translation | Participants described... |
| Participant perspective | Group represented | Youth, caregiver, clinician, teacher, other | Do not infer unreported identity | adolescents ages 15-17 |
| Context | Setting and construct | Structured plus text | Link to sample and module | school-based PSMU intervention |
| Negative case | Contradictory or disconfirming | Yes, no, unclear | Retain and flag | Yes |
| Source locator | Location of finding or quotation | Exact page, paragraph, table, quote ID | Required and audited exactly | p. 8, Theme 2, quotation 3 |
| Translation verification | Verification record | Human verifier, date, corrections | Required for non-English finding | Arabic original checked; one polarity correction |

## Secondary evidence and overlap matrix

Every included systematic review, meta-analysis, or scoping review will be linked in a citation matrix to the primary studies in its reference list. Eligibility is never inherited. A primary study enters the primary dataset only if independently retrieved and eligible. Secondary-review conclusions may suggest a possible gap, but no gap will be reported until the direct primary-evidence map is checked. Scoping reviews are charted separately from systematic reviews and receive descriptive PRISMA-ScR method fields rather than AMSTAR 2.

| **Matrix field** | **Definition** | **Decision rule** | **Hypothetical example** |
| --- | --- | --- | --- |
| review_id | Included secondary review | Stable identifier | REV000013 |
| Cited primary identity | DOI, title, authors, year | Verify identity before linking | 10.xxxx/example |
| Independently retrieved | Whether master search found record | Yes, no, unclear | Yes |
| Eligible primary source | Independent eligibility result | Include, exclude plus reason, pending | Exclude: age not extractable |
| Overlapping estimate | Same estimate as another report | Link result IDs; chart once | RES000912 |
| Gap implication | Potential versus verified gap | Potential, checked-no-primary, primary-present | primary-present |

## Author-contact rule

Contact is limited to eligibility-critical age data, identity of the target construct or instrument and cutoff, or a primary estimate essential to a prespecified output. Routine missing fields are NR. One request and one reminder after four weeks are permitted. Charting continues while a response is pending; nonresponse does not cause exclusion. Public logs contain non-identifying process data only.
