# Supplementary material for: Digital behavioral addictions in adolescents and young adults: Protocol for a scoping review of definitions, measurement, epidemiology, determinants, harms, interventions, and equity
Source: PLoS One. 2026 Sep 11;21(9):e0348860. doi: 10.1371/journal.pone.0348860 (PMC13567778; doi:10.1371/journal.pone.0348860)
Supplement: Supplementary file 4 [file pone.0348860.s004.docx]

# S4 Appendix. Protocol Amendment Log and Study Timeline

## Protocol amendment log

| **Date** | **Section or artifact** | **Registered or prior R1 position** | **Corrected position** | **Rationale** | **Implementation status** |
| --- | --- | --- | --- | --- | --- |
| 2026-04-21 | Abstract, registration line, data statement, S1 item 2, and prior timeline | DOI 10.17605/OSF.IO/39FTA | DOI 10.17605/OSF.IO/NPWTV | Canonical registration DOI was changed across the working package; the contemporaneous log called 39FTA "retired," a status statement corrected on 2026-07-12 | Historical amendment preserved; record-status language superseded by the 2026-07-12 clarification |
| 2026-04-21 | OSF registration title metadata | "Gaming Disorder And Digital Behavioral Addictions In Adolescents And Young Adults: A Scoping Review Protocol" | Current manuscript title | Aligned the public title with the submission title | Historical amendment preserved; live-record synchronization is rechecked in the 2026-07-12 update preview |
| 2026-04-21 | Study status and timeline | Quarterly milestones | PLOS Study Protocol monthly anchors for data collection and results | Addressed the initial technical request for specific timing | Historical amendment preserved; dates were re-anchored on 2026-07-01 and remain author-confirmation items |
| 2026-07-01 | S2 MEDLINE language limit | English, Spanish, or French database limit | Database language limit removed; language eligibility applied during screening | Reconciled the strategy with the seven-language eligibility criterion | Historical amendment preserved; the corrected R1 now searches without database-level language limits |
| 2026-07-01 | S2 OSF DOI | 39FTA link remained in S2 | NPWTV DOI inserted | Completed the then-planned cross-file DOI migration | Historical amendment preserved; distinct NPWTV and RAV8M roles are clarified on 2026-07-12 |
| 2026-07-01 | Measurement module and references | COSMIN v1.0 source paired with a v2.0 procedure | Mokkink, Elsman, and Terwee 2024 COSMIN v2.0 source | Corrected the edition mismatch | Historical amendment preserved and further operationalized in the corrected measurement form |
| 2026-07-01 | Study timeline | PRESS May-June 2026; search July 2026; completion May 2027 | PRESS July-August 2026; search September 2026; completion July 2027 | Re-anchored projections to the R1 resubmission period | Historical amendment preserved; final dates require author confirmation before upload |
| 2026-07-12 | Registration identifier | Prior working materials alternated between 39FTA and NPWTV and later called 39FTA retired | NPWTV is the immutable registration; RAV8M is the mutable project; 39FTA and UYBA2 are historical or superseded records | Distinguishes record roles without changing registration history | Manuscript and SI corrected; exact OSF sequence completed and anonymously read back 13 July 2026 |
| 2026-07-12 | Publication dates | Construct-specific eligibility dates from 2013, 2015, 2016, or 2018 | Search from inception; no publication-date exclusion; milestones charted as historical strata only | Earlier target-specific evidence may inform definitions, instruments, or history | Implemented in manuscript, S2, S3, Fig 1, and approved NPWTV update |
| 2026-07-12 | Search concepts | Construct, population, and mandatory domain block | Target construct or instrument AND population; no mandatory domain block | A domain block can miss definitions, qualitative, equity, and implementation evidence | Implemented in manuscript and S2; PRESS pending |
| 2026-07-13 | MEDLINE sensitivity and ages 25-29 | Video Games was the only addiction-related subject heading; free-text variants were narrower; Young Adult indexing did not cover ages 25-29 | Added current Internet Addiction Disorder indexing, legacy Behavior, Addictive paired with technology headings, broad dependence/overuse/dysregulation/maladaptive/pathological/problem-use variants, an un-exploded Adult retrieval route, and a prespecified age-stratified sentinel-recall gate | Aligns the draft with sensitivity-oriented guidance and protects retrieval of separately extractable 25-29 evidence | Implemented in manuscript and S2; PRESS and sentinel testing pending |
| 2026-07-12 | Targeted organization search | WHO, professional bodies, youth agencies, and relevant guideline developers were open-ended | Closed to WHO/ICD, APA/PsychiatryOnline, UNICEF, UNESCO, ITU, and NICE; six exact phrase site queries per listed domain; all results if 50 or fewer, otherwise first 50; no recursive crawling | Prevents discretionary source expansion and makes supplementary searching reproducible | Implemented in manuscript and S2; execution pending |
| 2026-07-12 | Population terms | Adolescent and young-adult terms centered on older youth | Added child, preadolescent, tween, and school terms | Makes ages 10-12 retrievable | Implemented in S2; PRESS pending |
| 2026-07-12 | Measurement eligibility | Contradictory exclusion of unvalidated measures and requirement for published validation | Per-use Tier 1, Tier 2, Tier 3, or N/A; no tier-based exclusion | Preserves breadth while preventing weak measures from being labeled diagnostic evidence | Implemented in manuscript and S3 |
| 2026-07-12 | Measurement-tier provenance | No reproducible process for locating validation evidence used in tier assignment | Two-reviewer lookup across source citations, manuals or developer sites, PubMed, PsycINFO, and COSMIN; exact searches, identifiers, population-language match, and decisions logged | Makes "no validation evidence identified" reproducible and auditable | Implemented in manuscript; validation log will be used during charting |
| 2026-07-12 | Prevalence terminology | Scale thresholds could be described as disorder prevalence | Diagnostic prevalence is reserved for appropriate diagnostic ascertainment; scale thresholds are screen-positive prevalence; Tier 3 is never disorder prevalence | Prevents diagnostic overstatement | Implemented in manuscript and S3 |
| 2026-07-12 | Generic Internet instruments | IAT and CIUS could establish eligibility alone | Eligible only when applied to a named target behavior with separately extractable findings | Excludes undifferentiated Internet overuse while retaining target-specific applications | Implemented in manuscript, S2, and S3 |
| 2026-07-12 | Core population | Mean or median age within 10-24 could establish eligibility | Core requires a wholly 10-24 sample or separately extractable subgroup; mean or median alone never establishes core evidence | Prevents age misclassification | Implemented in manuscript and S3 |
| 2026-07-12 | Ages 25-29 | Broad sensitivity group could include inseparable samples | Only separately extractable 25-29 data enter the secondary stratum; inseparable broad samples are contextual only | Protects primary age-specific interpretation and uses emerging-adulthood rationale cautiously | Implemented in manuscript and S3 |
| 2026-07-12 | Mixed-age samples | Mixed samples could contribute quantitative findings based on central age | Contextual evidence from mixed-age samples may contribute only definitions, instrument-use history, qualitative context, secondary-review context, or implementation description | Prevents false age-specific psychometric, epidemiologic, determinant, harm, equity, or effectiveness claims | Implemented in manuscript and S3 |
| 2026-07-12 | Languages | Registration listed English, Spanish, and French; R1 listed seven languages with a 10% machine-translation audit | English, Spanish, French, German, Portuguese, Arabic, and Hebrew; every final non-English decision and finding receives source-language human verification | Removes unsupported cross-language extrapolation and machine-only decisions | Implemented in manuscript and S3; human capacity documented 13 July 2026 and must be maintained |
| 2026-07-12 | Google Scholar | First 100 results per query and population wording centered on older youth | First 200 results per query; child, preadolescent, adolescent, youth, and young-adult terms; logged relevance order, date, time, browser/session, displayed count, and retained records | Aligns with Haddaway et al., retrieves ages 10-12, and improves reproducibility | Implemented in manuscript and S2 |

### Protocol amendment log (continued)

| **Date** | **Section or artifact** | **Registered or prior R1 position** | **Corrected position** | **Rationale** | **Implementation status** |
| --- | --- | --- | --- | --- | --- |
| 2026-07-12 | Charting workload and capacity | Workload was not quantified or tied to staffing | Thirty-two core fields; 12-26 fields per applicable module; calibration logs time, missingness, reconciliation, and assignment; charting pauses for amendment if dual-review capacity is inadequate | Converts broad-scope feasibility into measurable workload and a capacity contingency | Implemented in manuscript and response |
| 2026-07-12 | Result roles | Study-level determinant or harm role could duplicate constructs | Result-level temporality and direction hierarchy; undirected correlations stored once; reciprocal paths separated | Prevents contradictory or duplicate coding | Implemented in manuscript and S3 |
| 2026-07-12 | Multiple reports and reviews | Secondary evidence used descriptively without a complete overlap control | Study, report, sample, dataset-family, review, and result identifiers plus a citation matrix linking included reviews to their cited primary studies | Prevents inherited eligibility, double counting, and unsupported gap claims | Implemented in manuscript and S3 |
| 2026-07-12 | Qualitative synthesis | General descriptive thematic mapping | Versioned deductive-inductive codebook, dual coding of all findings, source locators, quotation-interpretation separation, negative cases, five-study calibration, and retrospective recoding | Increases reproducibility and preserves contradictory findings | Implemented in manuscript and S3 |
| 2026-07-12 | Appraisal | JBI plus modified Hoy for prevalence; AMSTAR 2 for all reviews; possible recommendation hierarchy | Exactly one source-level method by source level, primary aim, and primary design: relevant JBI checklists, MMAT, COSMIN RoB, AMSTAR 2, JBI systematic-review checklist, or PRISMA-ScR fields; unresolved classifications pause for prospective amendment | Removes duplicate appraisal of one source, closes unmapped-design gaps, and preserves the scoping/systematic boundary | Implemented in manuscript and S3 |
| 2026-07-13 | Calibration denominator | Agreement was limited to jointly chartable nonmissing categorical fields | NR, NA, and UNCLEAR are categorical decisions and mismatches count as disagreement; only jointly correct NA fields are excluded; critical fields with no applicable paired ratings are not passed and follow a confirmed deferral and reopening procedure | Prevents missingness disagreements from inflating the ≥90% and ≥80% gates | Implemented in manuscript, S3, and response |
| 2026-07-12 | Appraisal use | Appraisal could stratify or rank findings | No composite score, weighting, exclusion, certainty grade, or instrument recommendation hierarchy | Maintains a descriptive scoping-review purpose | Implemented in manuscript and S3 |
| 2026-07-12 | COSMIN rules | Simplified universal thresholds and traceability-free worked examples | COSMIN v2.0 property prerequisites and alternative criteria; every example is explicitly hypothetical | Aligns the form with current measurement guidance | Implemented in manuscript and S3 |
| 2026-07-12 | Author contact | Contact for any missing charting variable | Contact only for eligibility-critical or output-essential data; one request and one reminder; continue charting and code NR | Reduces burden and prevents workflow stalls | Implemented in manuscript and S3 |
| 2026-07-12 | Figure 1 | Fig 1 contained a date-exclusion reason | Fig 1 has two correct source lanes and no date exclusion | Synchronizes the selection template with the corrected search methods | Implemented and technically verified |
| 2026-07-12 | Data availability | NPWTV and RAV8M roles were conflated | Protocol-specific no-data statement; NPWTV is registration and RAV8M is the future-data project | Aligns with PLOS data policy and OSF record roles | Implemented in manuscript; EM identity check remains author action |
| 2026-07-13 | OSF record reconciliation | Corrected public text and files were approved but not yet executed | NPWTV update approved; dated corrected materials uploaded to RAV8M; historical notices added to 39FTA and UYBA2; exact fields, hashes, and states anonymously read back | Makes the public record consistent while preserving immutable and historical provenance | Completed; authenticated and anonymous readback verified |
